# Simultaneous measurement of p53:Mdm2 and p53:Mdm4 protein-protein interactions in whole cells using fluorescence labelled foci

**DOI:** 10.1038/s41598-019-54123-z

**Published:** 2019-11-29

**Authors:** Y. Frosi, K. Inoue, Siti Radhiah Ramlan, D. P. Lane, T. Watanabe, C. J. Brown

**Affiliations:** 1 p53 Laboratory, A*STAR (Agency for Science, Technology and Research), 8A Biomedical Grove, #06-04/05, Neuros/Immunos, Singapore, 138648 Singapore; 2 R&D Division, Medical & Biological Laboratories, Co., Ltd., 1063-103 Terasawaoka, Ina, Nagano, 396-0002 Japan

**Keywords:** Biochemistry, Biological techniques, Chemical biology, Drug discovery

## Abstract

In this report we describe the development of a **Flu**orescent **P**rotein-**P**rotein **I**nteraction-visualization (FLUOPPI) to enable the simultaneous measurement of both Mdm2:p53 and Mdm4:p53 interactions in order to assess the relative efficiencies of mimetic molecules of the p53 peptide helix against both PPIs. Mdm2 and Mdm4 overexpression frequently leads to the inactivation of non-mutated p53 in human cancers, via inhibition of its transcriptional activity, enhancing its degradation by the proteasome or by preventing its nuclear import. Development of inhibitors to disrupt the binding of one or both of these protein interactions have been the subject of intensive pharmaceutical development for anti-cancer therapies. Using the bimodal FLUOPPI system we have characterised compounds that were either monospecific for Mdm2 or bispecific for both Mdm2 and Mdm4. We have also demonstrated that the FLUOPPI assay can reliably differentiate between specific and non-specific disruption of these protein complexes via accurate assessment and normalization to the cell population under measurement. We envision that this methodology will increase the efficiency of identifying compounds that are either specific against a single PPI from a closely related family of interactions or compounds that interact across multiple related PPI pairs, depending on which is more desirable.

## Introduction

Intracellular protein-protein-interactions (PPIs) represent a wide class of potential drug targets across several important disease areas such as cancer and infectious diseases^1^. Over the last decade, large scale small molecule drug discovery has focussed on *in vitro* methods to discover new hit compounds that can disrupt specific protein-protein interactions (PPIs)^2^ such as Mdm2 and p53^3^. A large proportion of these protein-protein interactions form interfaces that are highly planar, not particularly hydrophobic and devoid of hydrophobic clefts^4^. Characteristics that make these interfaces intractable to traditional small molecule lead discovery approaches^4^. Small molecules only possess a relatively small surface area available for forming interactions with macromolecular surfaces, which is only maximized when they are bound in small clefts upon protein surfaces. This also makes them poor antagonists of PPIs that in contrast have much larger interaction surface areas^4^.

Antibodies and peptides constitute modalities that are much more efficient at disrupting PPIs than small molecules, as they have the capacity to form much larger interaction interfaces with their target molecules^5^. However, these larger molecular weight entities, unlike small molecules which can usually be designed to diffuse rapidly across the mammalian cell membrane, are not innately cell membrane permeable^5^. Many innovative approaches have been taken ranging from development of new chemically constrained peptidic entities to the design of delivery systems than can enable the intracellular penetration of impermeable cargo e.g. antibodies and scaffolds^6,7^. Currently extensive research is underway to identify new peptidic and non-peptidic modalities that can target disease relevant small molecule intractable PPIs^8,9^, such as KRAS and β-catenin. With the advent of new methodologies and chemistries to target these PPIs, the co-development of systems to confirm and validate engagement of the desired target and inhibition of its PPI within the cell are increasingly important^10–12^.

The use of a cell based assays over cell free biochemical and biophysical methods allows us to address issues such as cellular permeability and accessibility to subcellular organelles. Additionally, competitive interaction with other cellular factors and the effects of post-translational modifications can also be examined. Several different cell-based systems have been developed to measure the disruption of specific protein-protein interactions within live cells. These range from methodologies that utilize techniques such as fluorescent lifetime measurements^13^, fluorescence/bioluminescence resonance energy transfer (BRET)^14^, protein complementation assays (PCA)^15^, yeast two hybrid (Y2H)^16^ and cellular localization assays^10,11^. These methods are even more powerful with orthogonal measurements of viability and toxicity, which allow the specific effects of the compound acting on its target versus off-target and non-specific effects to be addressed. However, none of these methodologies have been extended to measure multiple interactions simultaneously. The quantitative measurement of molecules and their interactions with multiple PPIs would be advantageous as their specificity, off-target effects or poly-pharmacological^17^ properties could be assessed. Potentially this would allow the discovery and design of molecules with more tailored binding properties, and enable more efficient lead discovery to initiate therapeutic programs.

p53 is a key tumour suppressor protein, which primarily functions as DNA transcription factor, that is commonly abrogated in cancer^18^. p53 plays a crucial role in protecting cells from malignant transformation through the induction of cell cycle arrest, apoptosis or senescence^18^. A mechanism that frequently results in the inactivation of p53 is increased expression of the p53-negative regulators MDM2 and MDM4^19^. Both Mdm2 and Mdm4 attenuate p53 function either by inhibiting its transcriptional activity^20^, mediating its proteosomal degradation or by preventing its nuclear import^21,22^. However Mdm4, unlike Mdm2, has no intrinsic E3 ubiquitin ligase activity^23^. Instead Mdm4 forms heterodimeric complexes with Mdm2 whereby it stimulates the ubiquitin activity of Mdm2^23,24^. As a result p53 activity and protein levels are acutely suppressed by Mdm2 and Mdm4 overexpression. Development of inhibitors to disrupt the interactions of p53 with either Mdm2 or Mdm4, or both, are therefore highly desirable as they will prevent p53 degradation and restore a p53 dependent transcriptional anti-tumour response^18^.

p53 primarily interacts with both Mdm2 and Mdm4 via its intrinsically disordered N-terminal transactivation domain (TAD), which forms an α-helix when bound to the N-terminal p53 binding domain of either protein^25,26^. Both Mdm2 and Mdm4 show high degrees of sequence similarity to each other. The α-helix of p53 projects three critical residues (F19, W23 and L26) into a deep hydrophobic cleft upon the surface of either Mdm2 or Mdm4. A wide variety of small molecules (e.g. Nutlin^27^, AMG-232^28^, MI-773^29^) and other modalities (e.g. ATSP-7041^30^, sMTIDE-02^31^) have been discovered that mimic the interaction of this helix with Mdm2 and act as antagonists of p53 inactivation. However, a large majority of the small molecules developed exhibit little affinity and activity against Mdm4. Several Mdm2 specific molecules have entered initial clinical trials, but have been hindered by unwanted dose limiting toxicities in patients^32–34^.

Mdm4 possesses several distinct structural differences in the p53 peptide binding groove that causes a large majority of the small molecules that mimic these residues to bind Mdm4 very weakly^35^. Overexpression of Mdm4 in tumours has been demonstrated to compromise the efficiency of Mdm2 specific compounds, presumably through the maintenance of heterodimeric complexes of Mdm2 and Mdm4 that inhibit and target p53 for proteosomal degradation^36^. The importance of the reciprocal levels of Mdm2 and Mdm4 on transcriptional inhibition and degradation of p53, highlight the importance of targeting both proteins simultaneously to achieve efficient activation of p53 to achieve an optimal therapeutic response. Molecules ranging from stapled peptides (ATSP-7041^30^, sMTIDE-02^31^ and VIP-82^12^) to small molecules (RO-5963^37^) have been discovered and developed that inhibit both proteins. Currently clinical trials are ongoing with the dual Mdm2/Mdm4 stapled peptide (ALRN-6924), which has been reported to be tolerated well in patients as well as demonstrating anti-tumour activity^38^.

We therefore decided to develop the **Flu**orescent **PPI**-visualization (FLUOPPI^39^) to enable the simultaneous measurement of both Mdm2:p53 and Mdm4:p53 interactions in order to assess the relative efficiencies of mimetic molecules of the p53 peptide helix against both PPIs. We used the dual p53:Mdm2 and p53:Mdm4 PPI system to characterise compounds that were either monospecific for Mdm2 (Nutlin3a) or bispecific for both Mdm2 and Mdm4 (RO-5963). We predict that the application of this novel dual PPI live cell assay will enhance the process of identifying compounds that inhibit Mdm2 and Mdm4 and in turn activate p53. It is also envisioned that this methodology can be used to identify compounds that are either specific against a single PPI from a closely related family of interactions or compounds that interact across multiple related PPI pairs, depending on which is more desirable.

## Results and Discussion

### Developing a quantitative assay to measure p53-Mdm2 and Mdm4 disruption *in situ* using FLUOPPI

**Figure 1:**
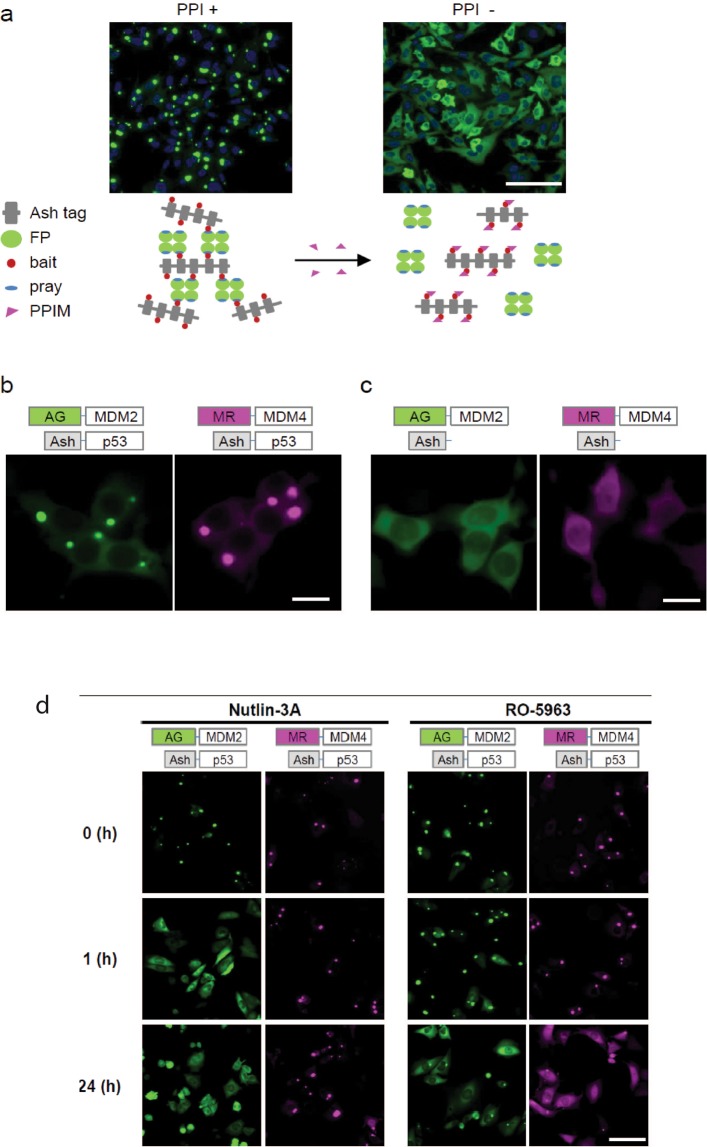
Schematic representation and construction of Fluoppi assay. (**A**) Mechanism of Action of Fluoppi. Ash tag and tetrameric fluorescence protein are fused to bait and prey proteins, respectively. Interaction between bait and prey (left panel, PPI+) proteins produces fluorescent foci inside mammalian cells via multivalent bait-prey interactions between single molecules. When the bait-prey protein interaction is inhibited by a PPI modulator (PPIM), FP tagged prey protein diffuse throughout the cells (Right panel, PPI−). Representative images of arbitrary Fluoppi expressing HEK293 cells are shown. Scale bar, 100 µm. **(B)** Representative images of selected Ash-p53:AG-Mdm2 and Ash-p53:MR-Mdm2 control pairs. Bright fluorescent foci were formed in the cytosol. **(C)** Representative images of selected Ash:AG-Mdm2 and Ash3:MR-Mdm4 negative control pairs. No Foci were detected with the negative Fluoppi control pairs, where either FP tagged Mdm2 or Mdm4 are co-expressed with the non-fused Ash tag by itself. **(D)** The reversibility of each of the systems were demonstrated either by treatment with an Mdm2 specific inhibitor (Nutlin-3A at 12.5 µM) or a dual inhibitor of both Mdm2 and Mdm4 (RO-5963 at 50 µM) at the following time points: 0 hour (pre-treatment), 1 hour or 24 hours. With Nutlin-3A, fluorescent foci mediated by the Mdm2:p53 interaction (green) but not the p53:Mdm4 (red) interaction were disrupted at both 1 hour and 24 hour treatment time points. RO-5963 only after 24 hours of treatment disrupted both sets of fluorescence foci meditated by both p53:Mdm2 or p53:Mdm4. Scale bar, 20 µm (**B**,**C**), 100 µm (**D**).

The FLUOPPI^39^ (MBL) technology is an imaged based assay system that offers several advantages over other plate based reading methodologies (e.g. protein complementation assays (PCA) and florescence resonance energy transfer (FRET)): (1) allows single cell analysis, (2) detect rare events and (3) enables the characterization of other cellular properties such as morphology and localization that can expedite the elimination of false positives. FLUOPPI^39^ utilises the formation of fluorescence foci whereby the interacting proteins of interest are genetically fused with either a tetramerizing fluorescent protein (FP-tag) or an assembly helper tag (Ash-tag). The incorporation of these tags onto a pair of interacting proteins (e.g. p53 and Mdm2) enable the formation of intensely bright foci when co-expressed in mammalian cells (Fig. 1A). In these foci the FP-tag induces the fused protein to form a tetramer that can now interact with up to 4 copies of the Ash-tagged partner protein. The cognate interacting protein also forms an oligomer through the Ash tag, which in turn can interact with multiple copies of the FP-fused partner protein. The potential of both constructs to interact with multiple copies of each other enable large foci incorporating the fluorescent protein to form, which can be clearly observed when imaging the cell. The integrity of these foci in cells depend on the interaction of the proteins fused to the Ash and FP tags, which makes the foci sensitive to antagonists of the PPI in question e.g. small molecule inhibitors or cellular signalling pathways (Fig. 1A). Upon disruption of the protein-protein interaction the fluorescence foci will disintegrate and diffusely stain the cell.

To develop an assay to simultaneously measure the interactions of both Mdm2 and Mdm4 with p53, a bimodal fluorescent based method was developed that utilised two different fluorescently tetramerizing tags (FP-tag), AG (Azami Green) with the Mdm2:p53 interaction and MR (Monti Red) with the Mdm4:p53 interaction. For the Mdm2:p53 PPI system all eight possible combinations of p53 (1–70) and Mdm2 (1–119) were fused to either the N or C terminal of the ASH or the AG (FP-tag) fluorescent protein, respectively. All 8 possible interactions pairs for the Mdm2:p53 PPI were then co-expressed in CHO-K1 cells and then analysed for foci formation 24 hours post-transfection (Fig. 1B). To eliminate the possibility of non-specific interactions between the expression constructs giving rise to foci formation, negative control experiments were performed between the AG fluorescent tagged constructs and the non-fluorescent oligomerizing tag (ASH) by itself (Fig. 1C). The Mdm2:p53 interaction pair (ASH-p53:AG-Mdm2) was then selected, which gave rise to discrete and intense foci that were uniformly present throughout the cell population and which also only exhibited a diffuse fluorescent stain in the relevant negative control experiment (Fig. 1B,C). Using this information, the p53 (1–70) and Mdm4 (1–112) PPI was then cloned and fused to the FP and ASH tags in the same orientation, with the fluorescence (FP) AG tag exchanged for the MR tag. The resulting p53 and Mdm4 PPI construct was then checked for the described criteria (Fig. 1B,C). This strategy was used as the structural interactions between Mdm2:p53 and Mdm4: p53 are remarkably similar in terms of structure and global orientation. The fluorescent foci for both interactions were exclusively localised in the cellular cytoplasm.

The reversibility of the p53:Mdm2 and p53:Mdm4 FLUOPPI systems were both tested and validated with the small molecules Nutlin 3A and RO-5963 to ensure that the fluorescent foci formed by both systems could be dissipated by disruption of their respective PPIs (Fig. 1D). Nutlin 3A is an Mdm2 specific inhibitor^27^ with a IC_50_ of 90 nM against Mdm2. The interaction of Nutlin 3A with Mdm4, the structural homologue of Mdm2, is greatly attenuated by approximately 100-fold^37^, due to structural differences in the p53 binding site. RO-5963 in contrast is a small molecule developed by Roche that is a dual inhibitor of both Mdm2 and Mdm4, and binds both molecules with IC_50_s of 17 nM^37^ and 24 nM^37^. Addition of either Nutlin 3A or RO-5963 (at respective concentrations of 12.5 µM or 50 µM) onto CHO-K1 cells transiently transfected with the ASH-p53:AG-Mdm2 PPI system for 24 hours, resulted in disruption of the fluorescent foci demonstrating that foci formation is reversible and dependent on the Mdm2:p53 interaction (Fig. 1D). When this experiment was repeated with the ASH-p53:MR-Mdm4 PPI system, foci disruption was only observed with the RO-5963 compound and not with the Mdm2 specific inhibitor Nutlin-3A, further showing that foci stability is dependent on the specific interactions of their fused interacting proteins (Fig. 1D).

### Measurement and quantitative assessment of the interactions of p53 with Mdm2 and Mdm4

**Figure 2:**
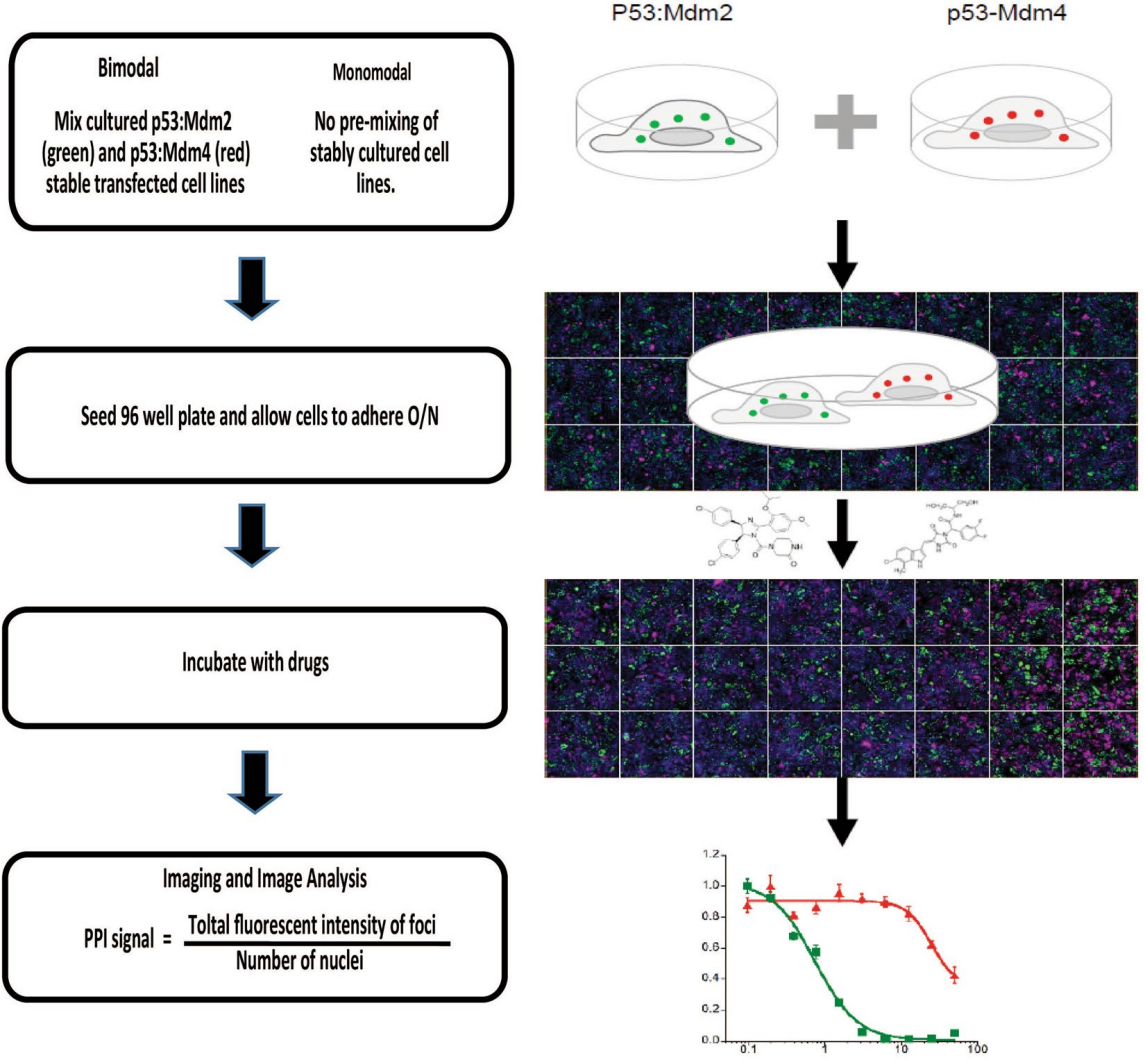
Workflow of Fluoppi bimodal p53:Mdm2 and p53:Mdm4 PPI system. Separate CHO-K1 lines stably expressing either Ash-p53:AG-Mdm2 or Ash-p53:MR-Mdm4 Fluoppi PPI pairs were established and maintained independently. To enable bimodal measurement both cell lines were pre-mixed in equal proportions and then used to seed 96 well plates for compound analysis. Stably transfected cell lines were used rather than transiently transfected cells in order to reduce experimental steps and to allow finer control over the ratio of Ash-p53:AG-Mdm2 to Ash-p53:AG-Mdm4 derived foci. Plates were incubated overnight and cells were treated with PPI modulating drugs. High content analysis equipment (HCA) was used to obtain images of green (Mdm2), red (Mdm4) and blue (Hoechest 33342) channels, which were then processed and segmented into nuclei and fluorescent foci using the Harmony High Content Imaging and Analysis Software (PerkinElmer). Cells were also processed using a nuclear morphology filter. This information was used to calculate the PPI (FLUOPPI) signal in accordance with the formula displayed. A minimum number of 500 foci were used to determine this value. Dose curves for each compound were then derived and used to calculate a corresponding IC_50_ value. A similar experimental setup can be used to analyse each stably transfected cell line separately.

To facilitate the development of a bimodal PPI assay, two independent cell populations stably transfected with the components required either for the p53:Mdm2 or p53:Mdm4 assays were generated (Fig. 2). To ensure that the amount of florescent foci in both the p53:Mdm2 and p53:Mdm4 stably transfected cell lines were as similar as possible, they were both generated from the same mono-stably transfected ASH-p53 containing cell line (Fig. S1). The presence of spatially distinct, intracellular and intensely bright fluorescent foci make the FLUOPPI system highly suitable for a high-content imaging (Fig. 2), which in turns allows quantitative IC_50_ determination and the screening of large compound collections (>1000).

**Figure 3:**
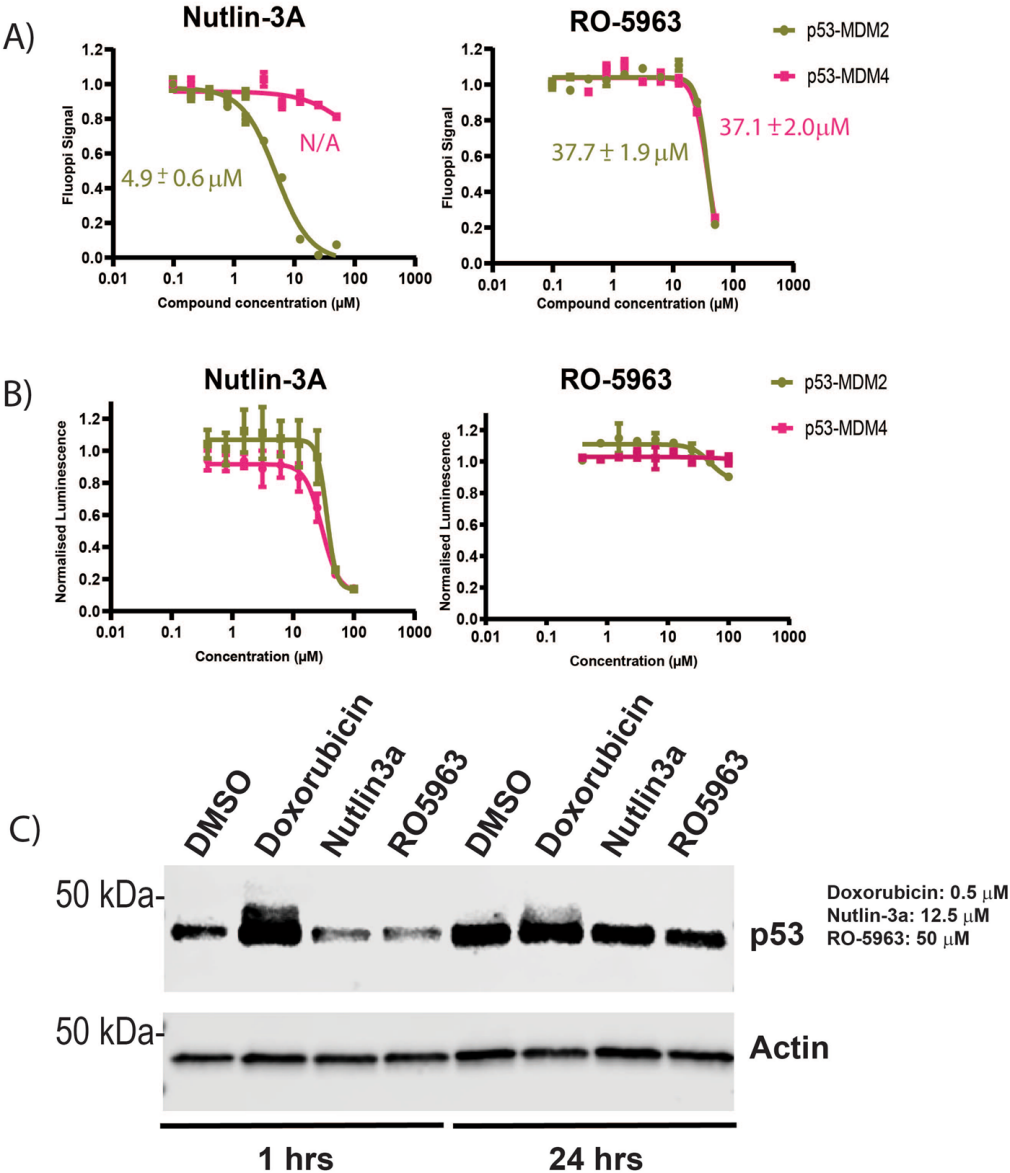
Validation of p53:Mdm2 and p53:Mdm4 Fluoppi systems. (**A**) Titrations of either Nutlin 3A or RO-5963 against unmixed cell populations of stably transfected CHO-K1 cells containing either the FLUOPPI p53:Mdm2 PPI or FLUOPPI p53:Mdm4 PPI system, respectively. Cells were treated with either Nutlin 3A for 1 hour or RO-5963 for 24 hours. Experiments were performed in DMEM cell media containing 10% (v/v) FCS. **(B)** Corresponding titrations to those performed in (B) were carried out to assess the effects of both compounds on the cell viability of either cell line (FLUOPPI p53:Mdm2 PPI or FLUOPPI p53:Mdm4 PPI). **(C)** Western blot analysis of endogenous p53 in un-transfected CHO-K1 cells treated with either doxorubicin, Nutlin 3A or RO-5963 for either 1 hour or 24 hours. Actin blot was used as loading control. For original unedited western blot images see supplementary information.

The p53:Mdm2 and p53:Mdm4 FLUOPPI stably transfected cell lines were validated independently from each other with titrations of Nutlin 3A or RO-5963 at 1 hour and 24 hours (Fig. 3A). Nutlin 3A disrupted the ASH-p53:AG-Mdm2 PPI system with an IC_50_ of 4.9 ± 0.6 µM, whilst at 24 hours RO-5963 inhibited both ASH-p53:AG-Mdm2 and ASH-p53:MR-Mdm4 PPI systems with values of 37.7 ± 1.9 µM and 37.1 ± 2.0 µM. Both compounds were also assessed for their effects on CHO-K1 cell viability, where only Nutlin 3A was observed to induce significant decreases in cellular viability. However, this was at a concentration 5-fold higher than its determined IC_50_ (Fig. 3B). To ensure that the responses in the p53:Mdm2/4 FLUOPPI PPI assays were due to direct target engagement by either Nutlin 3 A or RO-5963, CHO-K1 cells were assessed for their endogenous p53 response to both small molecules (Fig. 3C). Neither Nutlin 3A nor RO-5963 caused any observable stabilization or induction of p53 after 1 hr or 24 hours of treatment. These results demonstrate that the disruption of the fluorescent foci in the two FLUOPPI PPI assays is due to direct antagonism by both Nutlin 3A and RO-5963. Additionally, if Nutlin 3A had led to large increases in endogenous p53 protein levels in CHO-K1 cells disruption of the Mdm4:p53 fluorescent foci would have been expected as well. This was not observed (Fig. 3A).

### Simultaneous measurement of p53 with Mdm2 and Mdm4 in Live cells

**Figure 4:**
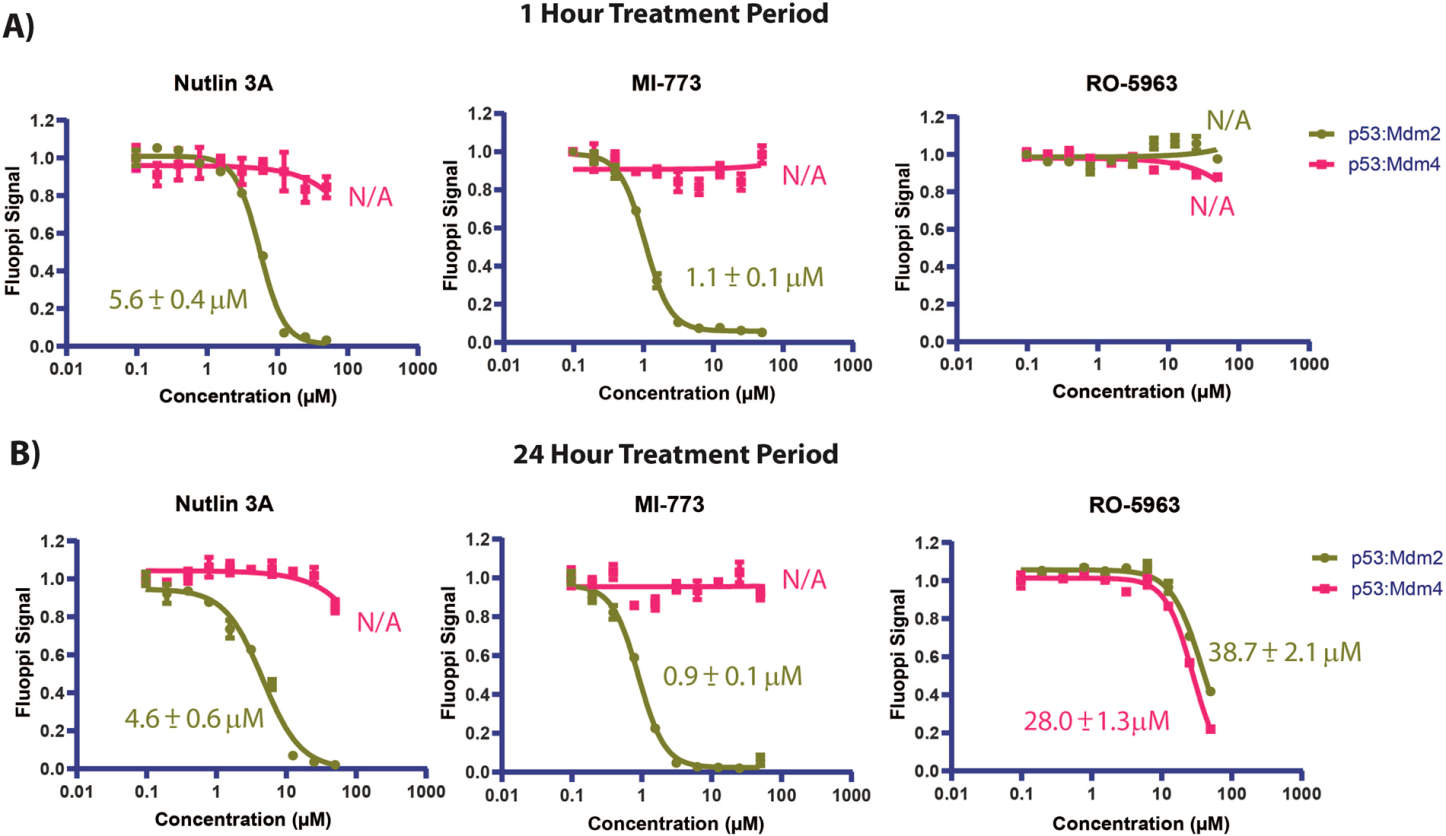
Titrations of the Mdm2 specific small molecule inhibitors Nutlin 3A and MI-773, and the small molecule Mdm2/Mdm4 dual inhibitor RO-5963, against the dual Mdm2:p53 and Mdm4:p53 FLUOPPI system at **(A)** 1 hour and **(B)** 24 hour time-points. IC_50_s were determined for each compound against each PPI (Mdm2:p53 and Mdm4:p53) using a 4 parameter model. Derived values are colour coded and labelled next to their respective fits. Curve fitting was performed using Prism 8 (Graphpad). Experiments were performed in DMEM cell media containing 10% (v/v) FCS.

Bimodal measurement of both p53:Mdm2 and p53:Mdm4 complexes in parallel was enabled through a cell pre-mixing (50:50) approach (Figs. 2 and 3). The image based dual Mdm2:p53 and Mdm4:p53 FLUOPPI cell based assay system was used to quantitatively re-determine the respective IC_50_ values for Nutlin 3 A and RO-5963 against both interactions (Fig. 4). IC_50_s determined against Mdm2 with Nutlin 3 A showed no significant change between treatment periods of 1 and 24 hrs with values of 5.6 ± 0.4 µM and 4.6 ± 0.4 µM, respectively. As expected Nutlin 3 A, which only efficiently interacts with Mdm2, showed negligible inhibition of the p53:Mdm4 FLUOPPI PPI. Additionally, the IC_50_ derived at 1 hour in the bimodal system is in close agreement with the IC_50_ determined for Nutlin 3A against the Mdm2:p53 PPI system, when cultured alone (Fig. 2B).

In contrast the RO-5963 compound, which is a potent inhibitor of both interactions of p53 with Mdm2 and Mdm4, exhibited no inhibition of either FLUOPPI PPI system after 1 hour of treatment. However, after a 24 hour exposure period of the CHO-K1^MR-Mdm4^ and CHO-K1^AG-Mdm2^ cells to RO-5963 a significant decrease in the FLUOPPI signal for both systems was observed (Fig. 4 and Table 1), revealing that RO-5963 inhibits Mdm2 poorly in comparison to Nutlin 3A. These values were also in close agreement with the IC_50_s generated in both PPI systems separately (Fig. 2B). This analysis was also expanded to include the Mdm2 inhibitor MI-773 (Fig. 4), a mono-specific inhibitor of the p53:Mdm2 interaction, which has been reported to be significantly more potent than Nutlin-3A using a fluorescence polarization assay with an IC_50_ of 0.88 nM versus 90 nM, respectively^29^. As with Nutlin 3A, MI-773 showed activity against Mdm2 at both time points with a corresponding negligible effect on the p53:Mdm4 interaction in the FLUOPPI PPI systems. The MI-773 compound is also approximately 5-fold more active than Nutlin 3A at both time points (Fig. 4).

**Table 1:** Comparison of IC_50_ values determined for various small molecule and stapled peptide inhibitors of Mdm2/Mdm4 in the following assays: (1) Mdm2/Mdm4:p53 bimodal FLUOPPI assay, (2) the Mdm2/4 and p53 NanoBIT PPI assays, (3) intracellular ATP determination assay (CellTiterGlow, PROMEGA) and (4) the LDH release assay (CytoToxOne, PROMEGA).

Compound	FLUOPPI Mdm2:p53 (IC50, µM)	FLUOPPI Mdm4:p53 (IC50, µM)	NanoBIT Mdm2:p53 (IC50, µM)	NanoBIT Mdm4:p53 (IC50, µM)	Viability (IC50, µM)	LDH Release (EC50, µM)
**Small Molecules**						
Nutlin 3A	5.6 ± 0.4/4.6 ± 0.6	NA/NA	1.6 ± 0.1/2.7 ± 0.4	40.5 ± 6.0/40.8 ± 7.2	34.0 ± 7.0/28.5 ± 4.7	35.7 ± 6.2/ND
MI-773	1.1 ± 0.1/0.9 ± 0.1	NA/NA	0.27 ± 0.01/0.25 ± 0.01	10.2 ± 1.6/2.7 ± 0.4	NA/23.2 ± 0.02	30.0 ± 6.2/ND
RO-5963	NA/30.0 ± 1.3	NA/38.7 ± 2.1	87.3 ± 18.4/41.4 ± 5.9	54.7 ± 9.3/12.5 ± 3.8	NA/NA	NA/NA
**Staple Peptides**						
VIP-82	38.0 ± 18.0/12.3 ± 1.9	20.6 ± 4.6/2.9 ± 0.2	3.0 ± 1.7/0.8 ± 0.1	1.2 ± 0.1/0.9 ± 0.1	NA/8.5 ± 1.1	NA/10.0 ± 1,0
VIP-82^SCRAM^	NA/49.9 ± 16.6	NA/NA	17.7 ± 8.9/12.7 ± 2.0	6.0 ± 0.9/4.5 ± 1.2	NA/12.3 ± 1.0	21.3 ± 2.7/10.1 ± 2.2

The first value quoted in each table cell refers to a compound treatment period of either 1 hours in the case of small molecules and 4 hours in the case of the stapled peptides. The second value in each cells refers to a compound treatment period of 24 hours. NA = No activity. ND = Not determined. IC_50_ values were not determined for titrations that sampled insufficient points for the derivation of accurate values. Table does not include IC_50_ values derived for normalised data. IC_50_s were also determined for Nutlin-3A against FLUOPPI Mdm2:p53 and Mdm4:p53 systems at 1 hour, independently of each system. These were 4.9 ± 0.6 µM and N/A, respectively. IC_50_s were also determined for RO-5963 against FLUOPPI Mdm2:p53 and Mdm4:p53 at 24 hour, seperately as well. These were 37.7 ± 1.9 µM and 37.1 ± 2.0 µM, respectively.

### Comparison of the bimodal FLUOPPI system to an alternative Protein complementation (PCA) derived PPI quantitation tool

To evaluate the advantages and disadvantages of the FLUOPPI p53:Mdm2/4 PPI system we compared it to the commercially available NanoBIT p53:Mdm2 PPI cell-based assay (PROMEGA)^40^. The nanoBIT system is a protein complementation system derived from the nanoLUC luciferase (PROMEGA), which consists of 2 components termed LgBiT (18KDa protein fragment) and SmBiT (11 amino acid peptide fragment) that have been optimised for minimal self-association and stability. When LgBiT and SmBiT are optimally fused to two interacting proteins, in this case full-length Mdm2 and full-length p53, they will be brought sufficiently close to each other that they will reform the active luciferase and generate a luminescence signal. In order to make a complete comparison with the bimodal FLUOPPI system, the Mdm4 protein was also cloned into an identical position to the Mdm2 fusion in the relevant construct of the nanoBIT PPI system and experimentally revalidated (Fig. S2).

**Figure 5:**
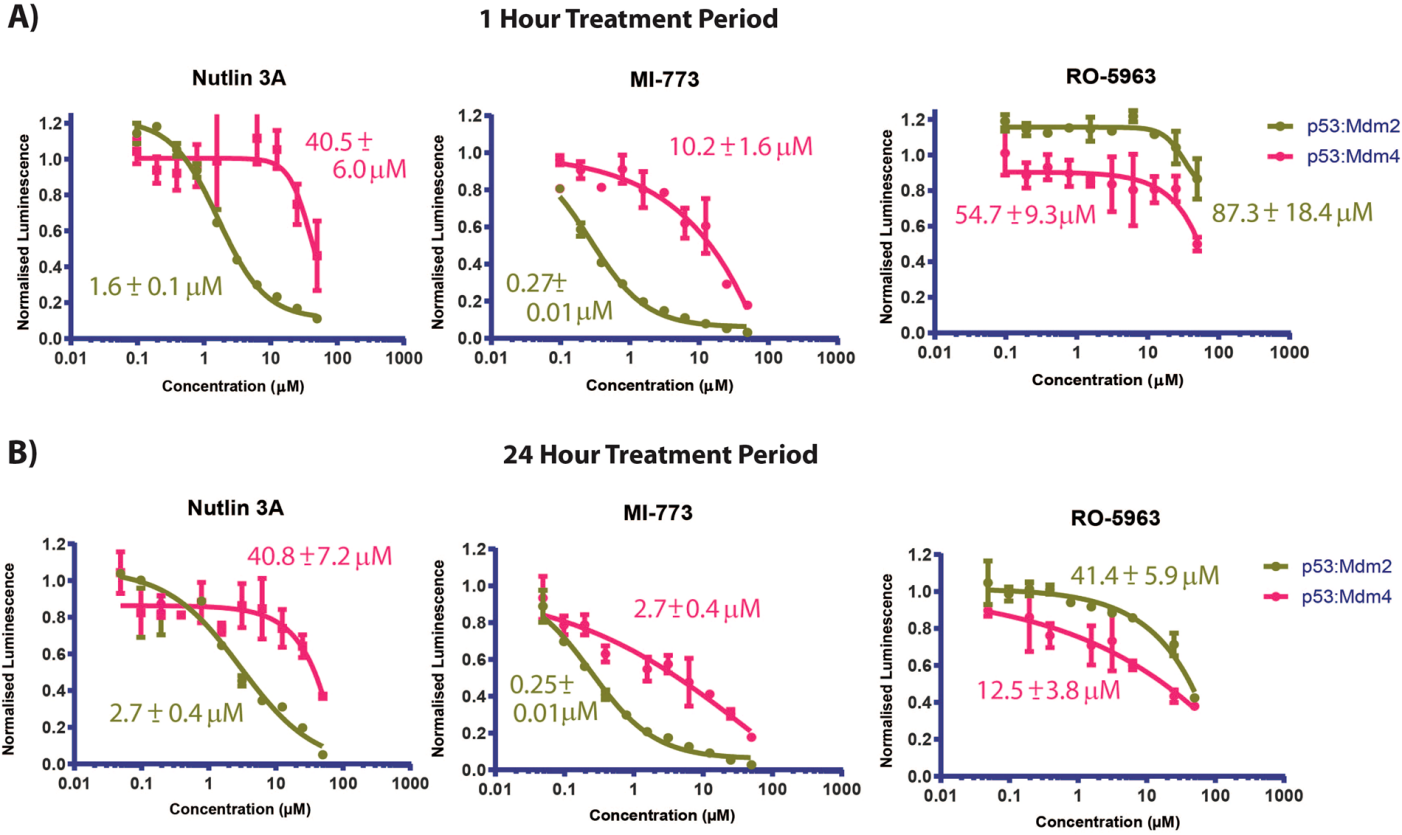
Titrations of the Mdm2 specific small molecule inhibitors Nutlin 3A and MI-773, and the small molecule Mdm2/Mdm4 dual inhibitor RO-5963, against CHO-K1 cells either transiently transfected with the Mdm2:p53 or the Mdm4:p53 NanoBIT system at **(A)** 1 hour and **(B)** 24 hour time-points, respectively. IC_50_s were determined for each compound against each PPI (Mdm2:p53 and Mdm4:p53) using a 4 parameter model. Derived values are colour coded and labelled next to their respective fits. Curve fitting was performed using Prism 8 (Graphpad). Experiments were performed in DMEM cell media containing 10% (v/v) FCS.

Unlike the bimodal FLUOPPI system, the NanoBIT p53:Mdm2 and p53:Mdm4 systems must be transfected into different cellular populations and measured independently of each other to assess the effects of small molecule inhibition on each PPI. The small molecules Nutlin 3A, MI-773 and RO-5963 were therefore titrated onto CHO-K1 cells, transiently transfected with either the Mdm2 or the Mdm4 NanoBIT PPI system, and their effects re-evaluated in comparison to the FLUOPPI system, after treatment periods of either 1 hour or 24 hours (Fig. 5). Nutlin 3A and MI-773 at both time points were much more potent in disrupting the p53:Mdm2 interaction than the p53:Mdm4 complex, with little variance in their IC_50_s between either time-point (Table 1). The IC_50_s in comparison to those derived from the FLUOPPI were also approximately 2 to 4 fold lower at both time-points. However, Nutlin 3A and MI-773 exhibited greater effects in the NanoBIT p53:Mdm4 PPI assay than in the FLUOPPI system at both treatment time points, which increased with prolonged incubation with either compound (Fig. 5 and Table 1). Both compounds also had significant effects on cellular viability (arbitrarily defined as >25%) at concentrations greater than 25 µM, which also correlated with the release of cytosolic lactate dehydrogenase (LDH, Figs. 7 and S3). These results indicate the non-specific effects of these compounds at these concentrations. If these points are eliminated from the cellular viability analysis and the remaining viability data used to normalise the NanoBIT titrations the effects of Nutlin 3A on the p53:MDM4 interaction are minimal, whilst MI-773 still disrupts the NanoBIT derived p53:Mdm4 biosensor in a dose responsive manner (Fig. S4).

The dual Mdm2/4 inhibitor, RO-5963, was also tested in both NanoBIT PPI assays, where inhibition was observed at both 1 hour and 24 hours (Fig. 5, Table 1). As with the FLUOPPI system more significant inhibitory effects were observed at the later point, however some inhibition was observed at 1 hour unlike the FLUOPPI bimodal system. At the later time point the IC_50_ determined for the p53:Mdm2 in the NanoBIT system was similar to the FLUOPPI value, while that derived for the p53;Mdm4 PPI was ~ 3- fold lower. RO-5963, unlike Nutlin 3A and MI-773 exhibited no effects on cellular viability of CHO-K1 cells and exhibited minimal LDH leakage.

### FLUOPPI accurately measures specific disruption of the p53:Mdm2 and Mdm4 interactions whilst accounting for Off-target effects

Several cell permeable hydrocarbon stapled peptides that interact with high affinity against both Mdm2 and Mdm4 have been described^30,31,41^. These peptides possess a hydrocarbon ‘staple’ modification that consists of 2 olefin bearing non-natural amino acids that have been covalently fused together by ring closing metathesis (RCM) to form an alkane bridge, in order to stabilize the helical structure of the unbound peptides. The hydrocarbon modification has been reported to increase proteolytic stability, enable cellular uptake and improve distribution in *in vivo* models of a variety of peptides^42^. To further test and validate the FLUOPPI bimodal p53:Mdm2 and p53:Mdm4 PPI system a representative dual binding Mdm2/4 interacting stapled peptide, VIP-82^12^, was selected and synthesised, as well as the corresponding control compound VIP-82^SCRAM 43^. The control stapled peptide was designed by scrambling the three critical residues strictly required for binding to Mdm2/4. Scrambling of only these residues in the peptide sequence ensured that the structural and physiochemical parameters of the peptide including its amphipathicity, a variable critical for cellular entry of stapled peptides, were minimally perturbed.

**Figure 6:**
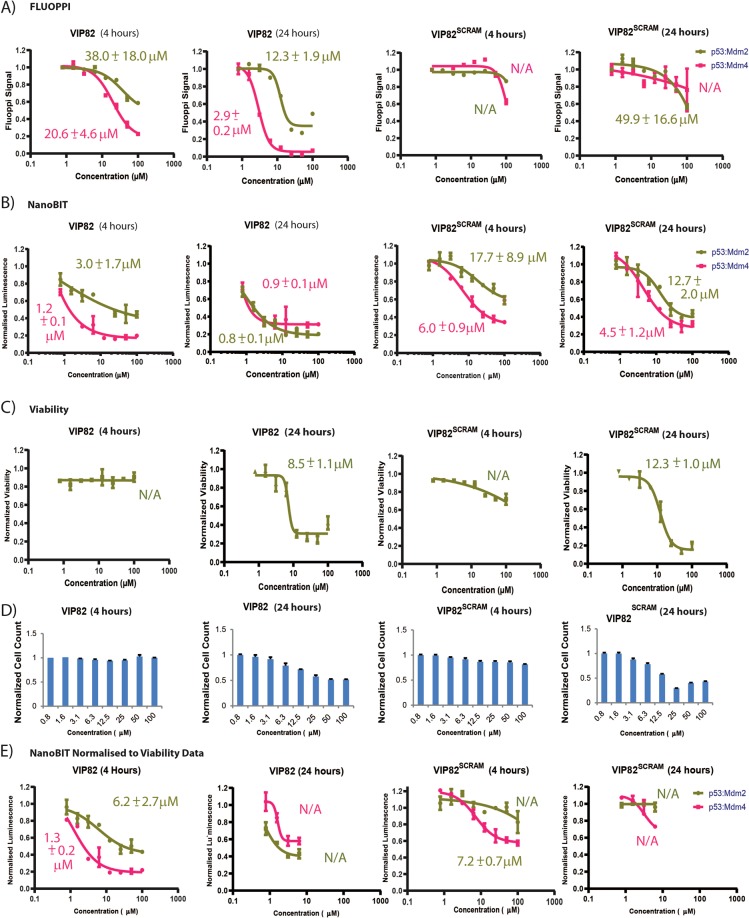
Titrations of the dual Mdm2/4 specific inhibitor VIP-82 and its scrambled negative control VIP-82^SCRAM^ against (**A)** CHO-K1 cells stably transfected with the bimodal FLUOPPI p53:Mdm2/4 system and (**B)** two independent cell populations either transiently transfected with the p53:Mdm2 nanoBIT system or the p53:Mdm4 nanoBIT system. Compound titrations were performed with either treatment periods of 4 hours or 24 hours. (**C)** CHO-K1 cells were treated with identical concentrations of VIP-82 and VIP-82^SCRAM^ as in (**A,B**) and their effects on viability were assessed at the same time points. (**D)** Bar charts indicate the relative number of cells used in the calculation of the FLUOPPI signal at each titration point indicated in (**A**). (**E)** Normalization of nanoBIT titrations results shown in B) to cell viability data in (**C**). Data points were removed at concentrations greater than 10 µM in the nanoBIT titrations normalized at 24 hours, due to substantial increases in luminescence values above the 1% DMSO v/v control. IC_50_ values are indicated next to the relative titration. IC_50_ values were derived from each individual titration using a 4-parameter curve model. Non-linear regression analysis to fit the curve was performed in Prism (Graphpad). Experiments were performed in DMEM cell media containing 2% (v/v) FCS

In the bimodal FLUOPPI, VIP-82 disrupted the interactions of both Mdm2 and Mdm4 with p53 with EC_50_s of 38.0 ± 18.0 µM and 20.6 ± 4.6 µM at 4 hours, and 12.3 ± 1.9 µM and 2.9 ± 0.2 µM at 24 hours, respectively (Fig. 6). However, unlike Nutlin 3A and MI-773, time dependent effects were observed for VIP-82 with a decrease in the determined IC_50_ values and an increase in the magnitude of total inhibition in the FLUOPPI signal against both PPIs at 24 hours. In the NanoBIT PPI assays, VIP-82 again disrupted both protein interactions at both time-points with IC_50_s of 3.0 ± 1.7 µM and 0.8 ± 0.1 µM against Mdm2, and 1.2 ± 0.1 µM and 0.9 ± 0.1 µM against Mdm4, respectively. However VIP-82^SCRAM^, unlike the FLUOPPI system where it had negligible effects on both PPIs, it induced significant inhibition in both Mdm2:p53 and Mdm4:p53 nanoBIT PPI assay systems. Additionally, the IC50 values determined for VIP-82 against both PPIs did not differ significantly between different compound incubation periods (4 hrs vs 24 hrs).

**Figure 7:**
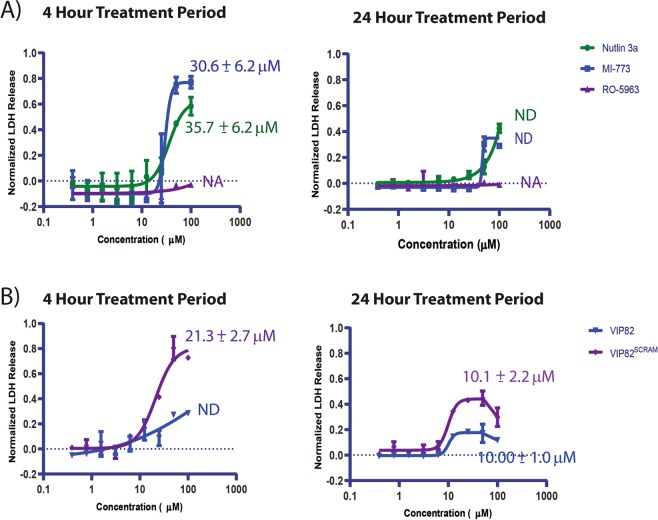
(**A)** Small molecules (Nutlin 3A, MI-773 and RO-5963) and (**B)** either VIP-82 or VIP82^SCRAM^ were titrated onto CHO-K1 cells for periods of either 4 or 24 hours and their resulting LDH profiles were assessed. Experiments were performed in DMEM media containing 2% FCS (v/v) with respect to the VIP-82 analogues and 10% in the case of the small molecules.

The IC_50_s determined for VIP-82^SCRAM^ were approximately 6-fold and 12-fold higher against Mdm2, and 6-fold and 5-fold higher against Mdm4 than those derived with VIP-82 at both time-points, implying that the inhibitory effects on the Mdm2:p53 and Mdm4:p53 nanoBIT complexes are not a direct consequence of Mdm2/4 inhibition. To further verify this hypothesis, both peptides were assessed for binding to Mdm2 and Mdm4 using a competitive fluorescence polarization assay, which confirmed that only VIP-82 and not the scrambled control peptide (VIP-82^SCRAM^) could interact with either protein (Fig. S5). We also assessed the cells for LDH leakage (CytoTOX, PROMEGA), a cellular cytoplasmic protein that is released from the cell upon membrane perturbation, and there viability as a function of intracellular ATP concentration (CellTiter-Glo, PROMEGA) (Figs. 6 and 7). VIP-82 and VIP-82^SCRAM^ lead to significant decreases in cellular viability at 24 hours, with IC50s^Viab^ of 8.5 ± 1.1 µM and 12.3 ± 1.0 µM, respectively, which were not observed at 4 hours. Significant release of LDH at 24 hours for both peptides was also seen (Fig. 7). However at the earlier 4 hour time-point, where both peptides had negligible effects on cell viability, VIP-82^SCRAM^ induced significantly greater amounts of LDH leakage than VIP-82 (Fig. 7) indicating it is destabilising the integrity of the cellular membrane and confirming that VIP-82 enters the cell more benignly.

VIP-82 mediated inhibition of Mdm2:p53 and Mdm4:p53 complex formation in the nanoBIT assays also occurs at substantially lower concentrations than its effects in the viability and LDH assays at 24 hours (Table 1), which is not the case for VIP-82^SCRAM^, where the derived IC50s in these assays are only 2-fold lower. These results indicate that at low peptide concentrations (approximate less than 10 µM), VIP-82 is having a real and specific inhibitory effect upon intracellular complex formation of Mdm2/4 with p53, whilst at higher treatment conditions, non-specific concentration dependent effects are occurring, as exemplified by VIP-82^SCRAM^, such as cell leakage and cell death that in turn causes misleading decreases in the nanoBIT signal. Results at 4 hours further support these conclusions with the LDH_50_ value calculated for VIP82^SCRAM^ almost identical to the NanoBIT Mdm2:p53 IC_50_ value (21.3 ± 2.7 vs 17.7 ± 8.9), whilst the VIP-82 peptide disrupts the NanoBIT Mdm2:p53 PPI assay with significantly reduced effects on LDH release (Table 1). However, VIP-82^SCRAM^ does appear to disrupt the nanoBIT Mdm4:p53 PPI system with an IC_50_ value below the threshold at which LDH release is observed. A putative mechanistic explanation for this behaviour is that the scrambled peptide maybe undergoing aggregation and having deleterious and p53 independent effects upon cellular mechanics after entry into the cell, whilst at higher concentrations it is able to efficiently disrupt the integrity of the cell membrane.

The increase in non-specific effects observed in the NanoBIT assay compared to the bimodal FLUOPPI assay principally arise from differences in assay output measurement and result analysis. In the NanoBIT assay the luminescent signal measurements originate from assessment of the whole well volume of the individual samples on the microplate, whilst in the bimodal FLUOPPI system only the cells attached to the floor of the 96 well plate are analysed to calculate the FLUOPPI signal (Fig. 3). Another key difference is that the bimodal FLUOPPI assay also uses a nuclear morphology filter to exclude the measurement of any adherent abnormal cells from the FLUOPPI signal calculation. These key differences between the two technologies in effect allows the FLUOPPI technology to measure only healthy cells and in turn accurately normalise the experimental signal to the number of cells under measurement. This is clearly demonstrated by the strong correlation between the cellular viability data and the total number of cells used to determine the FLUOPPI signal (Fig. 6E). This contrasts to the NanoBIT system that does not differentiate between cells within the population under measurement and also does not perform any internal normalization procedure. This can be overcome to a certain extent by normalising to another orthogonal cell measurement that determines viable cell number e.g. viability assay such as CellTiterGLow (PROMEGA) (Fig. 6D). However at high treatment concentrations, where cellular viability can be marginal, normalization can result in substantially larger luminescence values than those in the negative control (1% DMSO v/v) (Fig. 6E). Such points are usually removed for IC_50_ derivation as cell viability is serving as a poor proxy for the number of cells generating the NanoBIT signal.

## Conclusion

The ability to measure the effects of molecules across multiple PPIs (Protein-Protein Interactions) simultaneously, offers a dramatic improvement in the conservation of time and resources required in the initial stages of the hit to lead discovery process. The quantitative measurement of hit molecules and their effects on multiple PPIs would be advantageous as key protein-protein interactions could be included or excluded from the desired inhibition profile, in order to identify compounds with minimal on/off target toxicity or to eliminate potential innate chemo-resistance mechanisms from developing. The long term implications of such an approach may result in decreased attrition rates of lead molecules through lead development and clinical assessment.

In these studies we have developed a bimodal fluorescence foci PPI (FLUOPPI) based assay to measure the integrity of the interaction of p53 with both Mdm2 and Mdm4 in live cells. We have successfully demonstrated that the dual FLUOPPI PPI system can accurately measure the effects of Mdm2 specific (Nutlin 3A, MI-773) and bispecific (RO-5963) small molecule inhibitors on the p53:Mdm2 and p53:Mdm4 interactions pairs, respectively. We also used the dual FLUOPPI PPI system to assess compounds representative of a rapidly emerging class of compounds termed stapled peptides designed to inhibit both Mdm2 and Mdm4. A representative stapled peptide (VIP-82) was analysed and demonstrated to disrupt both sets of interactions. These results demonstrate that a multimodal PPI interrogation system, in relation to p53 and its two key negative regulators (Mdm2 and Mdm4), can be used to identify specific inhibitors of both Mdm2 and Mdm4 to overcome the antagonistic effects of uninhibited Mdm4 and any associated Mdm4 toxicities.

Several stapled peptides and conjugated CPPs (cell penetrating peptides) have been reported to have adverse structural effects on the integrity of lipid bilayers and cell membranes. Such effects can lead to the inadvertent entry of the molecule of interest into the cell and disruption of the PPI under study that do not reflect true cellular permeability. Additionally this phenomena can also lead to cell death and cellular stress responses that give rise to further false positives in other assays types e.g. gene reporters, viability assays and phenotype based assays. The ability of the FLUOPPI assay to measure the effects of putative inhibitors against a specific section of the cell population (as determined by adherence and nuclear morphology) more robustly than other assays, such as the nanoBIT, allows it to differentiate the non-specific effects of VIP-82 ^SCRAM^ from VIP-82 making it a very useful tool in establishing if compounds are mediating their effects through their expected modes of binding.

The IC_50_s determined between both the FLUOPPI and NanoBIT systems were overall in reasonable agreement with each other. However, it is interesting to note that the IC_50_ values derived for VIP-82 are considerably lower in the NanoBIT assays than in the bimodal FLUOPPI system (Table 1), and that this may arise from differences in their intrinsic interactions. For example, the FLUOPPI foci are higher order multimeric structures that may impede access of VIP-82 by acting as a molecular sieve. This contrasts to the binary complexes of the p53:Mdm2/4 NanoBIT system that are more accessible. Additionally, the FLUOPPI foci may also behave as liquid phase compartments^39^, which could affect how compounds with different physiochemical parameters partition into them (i.e. stapled peptides vs small molecules) and disrupt their cross-linking interactions.

The p53:Mdm2 and p53:Mdm4 interactions are an example of a relatively small group of interactions that have been successfully targeted successfully using both classical small molecule approaches and methodologies utilizing new modalities (e.g. stapled peptides). However, there is an enormous diversity of medically important PPIs that have not yet been successfully targeted and are considered much more intractable to small molecule discovery. As a result many alternative modalities are being developed to target these interactions, and it is increasingly being realised that demonstrating that these molecules directly engage their targets within live cells in the absence of unwanted side-effects is required to demonstrate their cellular permeability. The factors that have driven this requirement range from peptide driven gross membrane disruption and lysis, peptide aggregation and possible transient interactions of the peptide with cellular membranes leading to non-specific biological effects.

Due to the use of two independent stably transfected cell lines, differential effects of drug treatment upon either cell population may induce cellular effects that the bimodal FLUOPPI system may not capture. For example drug A may induce apoptosis in one cell line and the release of intracellular factors may indirectly cause FLUOPPI inhibition in the second cell line, or drug B may cause cell arrest in one of the two cell populations and over a long treatment period this will affect the relative number of cells from each population that are exposed to drug, which in turn could affect their relative IC_50_ determinations. Such effects could be caused by the presence of the different FLUOPPI complexes in either cell line i.e. release of the ASH-p53 component of FLUOPPI from only Mdm2. However, these types of differential effects are much more likely to occur if the two cell populations come from very different genetic origins, which is not the case for p53:Mdm2/4 system, as both cell lines originate from the same parent cell line that expressed the ASH-p53. However, the bimodal FLUOPPI assay is expected to be used as a primary screening tool, and if such differential effects are occurring then secondary assays (such as FLUOPPI in mono-modal format or other assays of specific biological function) should be run to verify their occurrence. Additionally, compounds that disrupt FLUOPPI foci (also applicable to the NanoBIT PCA assay) would usually be expected to induce endogenous p53 and Mdm2, unlike RO-5963 and Nutlin-3A in the case of the CHO-K1 cell line. Therefore, another pertinent counter-assay would be to perform the FLUOPPI assay (or the NanoBIT) transiently in a p53 knock out cell line to confirm direct Mdm2/4 engagement.

The modular FLUOPPI system opens up a range of possibilities whereby cell lines stably transfected with alternative PPIs can be mixed and matched to enable innovative screening campaigns for novel lead compounds. Currently, we have only utilized two compatible fluorophores (green, red), however this could be extended to further fluorophores to enable higher dimensional screening (cyan, far red) of other PPI interactions. This could also include the use of other dyes and visual descriptors related to desirable phenotypic outputs e.g. caspase activity, DNA fragmentation, allowing the advantages of an image based readout to be maximised. We envision that the potential versatility of the FLUOPPI PPI system can be applied to a wide range of lead discovery projects. For example, identifying compounds that are selective for mutant KRAS with little affinity for wild-type KRAS and its closely related homologues H-RAS and N-RAS would be highly desirable in terms of efficacy and avoidance of deleterious toxic effects. Additionally, the FLUOPPI assay will also useful in identifying novel chemical matter and cellular delivery methods for the efficient disruption of medically important protein: protein complexes.

## Methods and Materials

### FLUOPPI plasmids construction and cloning

See Supplementary Information.

### Cloning and Construction of nanoBIT MDM4-p53

See Supplementary Information.

Cell culture and transfection (including Transient FLUOPPI Transfection Experiments and FLUOPPI Stable Cell Line Generation).

See Supplementary Information.

### NanoBIT PPI assay transfection conditions

For transient transfection experiments, 24 hours prior to transfection CHO cells were seeded at a cell density of 300,000 or 1200,000 cells respectively per well of a 6 well plate (ThermoFisher Scientific). Each well was then transfected with LgBIT-p53 and either SmBIT-Mdm4 or SmBIT-Mdm2 plasmids in a ratio of 1:3 or 1:1 respectively by using Lipofectamine 3000 (ThermoFisher Scientific) according to manufacturer’s instructions. After a 24 hour incubation, medium was removed and cells were washed with PBS saline. Transfected CHO-1 cells were trypsinised and re-suspended in Opti-MEM media with 0% FCS. Cells were then spun down at 1000 rpm for 5 minutes at room temperature. Supernatant was then discarded and cells re-suspended to a density of 88000 cells per ml in Opti-MEM I reduced serum containing 0% FCS or 2% FCS with no added red phenol. 90 μl of the resulting cell suspension was added to the wells of a white opaque 96-well plate and incubated with either 10 μl of a 10% (v/v) DMSO control in FPLC grade water or a suitable 2-fold dilution series of the compound under study in a 10-fold higher stock concentration resuspended in 10% DMSO (v/v) in FPLC grade water solution. After 4 or 24 hrs of incubation with indicated compounds, plate were processed. 25 μl of a 20-fold dilution of Nano-Glo live cell substrate (Promega) in Nano-Glo dilution buffer (PROMEGA) was added to each well and the plate shaken for 1 min at 22 °C and luminescence assessed after additional 50 minutes as described previously^43^. All luminescence assay were carried out in triplicate and luciferase activity for each well was measured by Envision Multilabel plate reader (PerkinElmer). Curve-fitting was carried out using Prism 4.0 (GraphPad).

### Cell viability and LDH measurements

Cell viability was determined as indicated by the manufacturer’s instruction (CellTiter-Glo 2.0, Promega). Lactate dehydrogenase (LDH) release was assayed on medium supernatant of treated transfected cells as described by the manufacturers (CytoTox 96 Non*-Radioactive Cytotoxicity Assay*, Promega). Maximal LDH release was defined as the amount of LDH released when cells were lysed in the presence of 0.1% TRITON X-100 and was used for results normalization.

### FLUOPPI high-content imaging cell analysis

Two stable cell lines, 1A7-1 and 1B7-11 were mixed equally and seeded into poly-D-lysine black-wall 96-well plates (Corning) at a density of 20,000 cells/well and incubated for 24 hours at 37 °C in a 5% CO_2_ atmosphere. Culture medium were replaced with with indicated concentration of small molecules or stapled peptides diluted in DMEM containing the specified concentration of FBS. Final concentration of DMSO was 1%. Cells were treated with the compounds at 37 °C in a 5% CO_2_ atmosphere for indicated durations followed by staining with Hoechst33342 (Dojindo) or Draq5 (Abcam) for 30 minutes. Independent plates were used for different treatment periods. Plates were only removed from tissue culture incubation (37 °C, 5% CO_2_ v/v) prior to image acquisition. Image acquisitions were performed using Operetta high-content imaging system (PerkinElmer) with a 20 × objective lens, under normal atmospheric conditions (approx. 20–25 °C). Images of nine fields per well were acquired in triplicate. Image analysis was performed using Harmony software (PerkinElmer). Green (AG), red (MR) and blue (Hoechst33342) or far-red (Draq5) fluorescence images were used to monitor p53-MDM2, p53-MDM4 and nuclei, respectively. Puncta and nuclei were segmented automatically using “Find Spot” and “Find Nuclei” algorithms, respectively. To remove dead cells from analysis, only cells with a “nuclei roundness” score of more than 0.75 were counted. FLUOPPI values were calculated by dividing the total green or red fluorescence intensity of puncta by the number of AG-positive nuclei or MR-positive nuclei, respectively. The average FLUOPPI values were normalized to the ones at the lowest concentration of each compound. Curve fitting was performed using Prism 8 (Graphpad).

### Curve fitting procedures for FLUOPPI, NanoBIT, LDH and cell viability titration curves

See Supplementary Information.

### Mdm2/4 purification

See Supplementary Information.

### Competitive fluorescence anisotropy assays (Mdm2 and Mdm4)

See Supplementary Information.

### Peptide synthesis

See Supplementary Information.

## Supplementary information

Supplementary Information
