# Supplementary material for: Simultaneous measurement of p53:Mdm2 and p53:Mdm4 protein-protein interactions in whole cells using fluorescence labelled foci
Source: Sci Rep. 2019 Nov 29;9:17933. doi: 10.1038/s41598-019-54123-z (PMC6884555; doi:10.1038/s41598-019-54123-z)
Supplement: Supplementary file 1 — Supplementary Information [file 41598_2019_54123_MOESM1_ESM.docx]

**Supplementary Information**

**FLUOPPI plasmids Construction and Cloning**

FLUOPPI p53-MDM2 pair Ash-p53 and AG-Mdm2 were purchased from MBL. Human Mdm4 partial sequences was inserted into pMontiRed-MCLinker plasmid (MBL) via BamHI/NotI cloning sites in order to preserve similar linker length as for AG-Mdm2 construct.

**Cloning and Construction of nanoBIT MDM4-p53**

NanoBIT PPI control pair LgBIT-p53 and SmBIT-Mdm2 or SmBIT-Halotag were purchased from Promega. Human full-length Mdm4 coding sequence was inserted into SmBIT plasmid via NheI/EcoRI cloning sites in order to preserve similar linker length as for SmBIT-Mdm2 construct.

**Cell culture and transfection**

*Transient FLUOPPI Transfection Experiments*

24 hours prior to transfection CHO-K1 cells were seeded at a cell density of 800,000 cells respectively per well of a 6 well plate (ThermoFisher Scientific). Each well was then transfected with Ash-p53 and either MR-Mdm4 or AG-Mdm2 plasmids in a ratio of 1:1 respectively by using FuGeneHD (Promega) according to manufacturer’s instructions. After a 24 hour incubation, medium was removed and cells were washed with PBS saline. Transfected CHO-K1 cells were trypsinised and re-suspended in Nutrient Mixture F-12 Ham (Ham's F-12) media (Sigma-Aldrich) supplemented with 10% FCS. Cells were then spun down at 1000 rpm for 5 minutes at room temperature. Supernatant was then discarded and cells re-suspended to a density of 200,000 cells per ml in Ham's F-12 media supplemented with 10% FCS. 100 μl of the resulting cell suspension was added to the wells of a black clear bottom 96-well plate. After a 24 hours incubation, the cell medium was replaced with fresh Ham's F-12 medium containing the indicated concentration of small molecule or stapled peptide and the stated FCS concentration. Final concentration of DMSO was 1% (v/v).

*FLUOPPI Stable Cell Line Generation*

Chinese hamster ovary (CHO) cells were grown in Nutrient Mixture F-12 Ham (Ham's F-12) (Sigma-Aldrich) supplemented with 10% FBS and 1% penicillin/streptomycin (Life Technologies). Cells were incubated at 37 °C in a humidified atmosphere containing 5% CO2. To establish a cell line stably expressing PB1-p53, transiently transfected cells by FuGENE HD (Promega) were cultured for 2 weeks in selection medium containing 1,000 μg/mL of Geneticin (Gibco) followed by selection of single clone by limited dilution. Vectors encoding AG-MDM2 or MR-MDM4 with hygromycin resistance gene were transfected to the established stable cell line expressing PB1-p53. They were cultured for 2 weeks in selection medium containing 250 μg/mL of Hygromycin B (Thermo Fisher Scientific). Single clones of PB1-p53 and AG-MDM2 stably expressing cells (1A7-1) and PB1-p53 and MR-MDM4 stably expressing cells (1B7-11) were obtained by limited dilution.

**Curve Fitting Procedures for FLUOPPI, NanoBIT, LDH and Cell Viability Titration Curves**

IC_50_ and EC_50_ (with respect to LDH Release assays) were determined by fitting the relevant titration data to the 4 parameter logistic regression equation shown below:


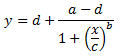


*a* is the response a zero analyte concentration, *d* is the response at infinite analyte concentration, *c* is the inflection point (EC_50_/IC_50_), *b* is the slope factor and variable *x* is the concentration of the analyte being varied during the experiment. Curve-fitting was carried out using Prism 4.0 (GraphPad). *a* was always constrained to the experimental value determined using the 1% DMSO (v/v) experimental control. After curve fitting the value determined for *d* was then verified to be in close agreement with the plateau of the titration. If the titration did not reach a defined plateau, *d* was constrained to an arbitrary value of 0.1 in the NanoBIT, FLUOPPI and viability assays. In the case of the LDH assay *d* was set to the lytic control (Triton X-100, 1% v/v).

**Mdm2 and Mdm4 protein expression and purification**

Mdm2 and Mdm4 purification. Mdm2 (1–125) and Mdm4 (1-125) were ligated into the GST fusion expression vector pGEX6P-1 (GE Lifesciences) via a BAMH1 and NDE1 double digest. BL21 DE3 competent bacteria were then transformed with the GST tagged (1–125) Mdm2 and Mdm4 fusion constructs. The cells expressing the GST fusion constructs were grown in LB medium at 37°C to an OD600 of ~0.6 and induction was carried out with 1 mM at room temperature. Cells were harvested by centrifugation and the cell pellets were resuspended in 50 mM Tris pH 8.0, 10% sucrose and then sonicated. The sonicated sample was centrifuged for 60 mins at 17,000 g at 4°C. The supernatant was applied to a 5 ml FF GST column (Amersham) pre-equilibrated in wash buffer (Phosphate Buffered Saline, 2.7 mM KCL and 137 mM NaCL, pH 7.4) with 1mM DTT. The column was then further washed by 6 volumes of wash buffer. Mdm2 and Mdm4 were then purified from the column by cleavage with Precission (GE Lifesciences) protease. 10 units of precission protease, in one column volume of PBS with 1mM DTT buffer, were injected onto the column. The cleavage reaction was allowed to proceed overnight at 4°C. The cleaved protein was then eluted of the column with wash buffer. Protein fractions were analyzed with SDS page gel and concentrated using a Centricon (3.5 kDa MWCO) concentrator, Millipore. Mdm2 and Mdm4 protein samples were then dialyzed into a buffer solution containing 20mM Bis-Tris, pH 6.5, 0.05M NaCl with 1mM DTT and loaded onto a monoS column pre-equilibrated in buffer A (20mM Bis-Tris, pH 6.5, 1mM DTT). The column was then washed in 6 column volumes of buffer A and bound protein was eluted with a linear gradient of 1M NaCL over 25 column volumes. Protein fractions were analyzed with SDS page gel and concentrated using a Centricon (3.5 kDa MWCO) concentrator, Millipore. The cleaved Mdm2(1-125) and Mdm4(1-125) constructs were purified to ~90% purity. Protein concentration was determined using A280 with extinction coefficients of 10430 M-1 cm-1 and 7575 M-1 cm-1 for Mdm2 (1–125) and Mdm4 (1– 125) respectively.

**Competitive Fluorescence Anisotropy Assays (Mdm2 and Mdm4)**

Purified Mdm2 (1-125) protein was titrated against 50 nM carboxyfluorescein (FAM)-labeled 12/1 peptide13 (FAM-RFMDYWEGL-NH2). Dissociation constants for titrations of Mdm2 and Mdm4 against FAM-labeled 12/1 peptide were determined by fitting the experimental data to a 1:1 binding model equation shown below:

Equation 1:


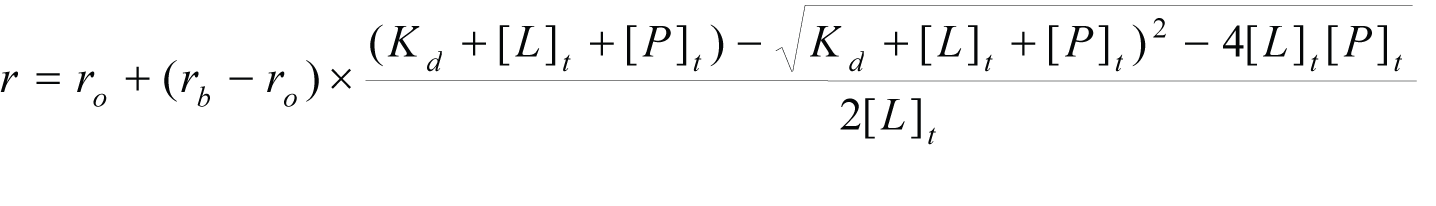


[P] is the protein concentration (Mdm2), [L] is the labeled peptide concentration, r is the anisotropy measured, r_0_ is the anisotropy of the free peptide, r_b_ is the anisotropy of the Mdm2–FAM-labeled peptide complex, K_d_ is the dissociation constant, [L]_t_ is the total FAM labeled peptide concentration, and [P]_t_ is the total Mdm2 concentration. The apparent K_d_ values for FAM-labeled 12/1 peptide against Mdm2 and Mdm4 were determined to be 13.0 nM and 4.0 nM, respectively. These values were then used to determine apparent K_d_ values of the respective competing ligands in subsequent competition assays in fluorescence anisotropy experiments.

Mdm2 and Mdm4 competition experiments were performed with their respective concentrations held constant at 250 nM and 75 nM, in the presence of 50 nM of FAM-labeled 12/1. The competing molecules were then titrated against the complex of the FAM-labeled peptide and protein. Apparent Kd values were determined by fitting the experimental data to the equations shown below:


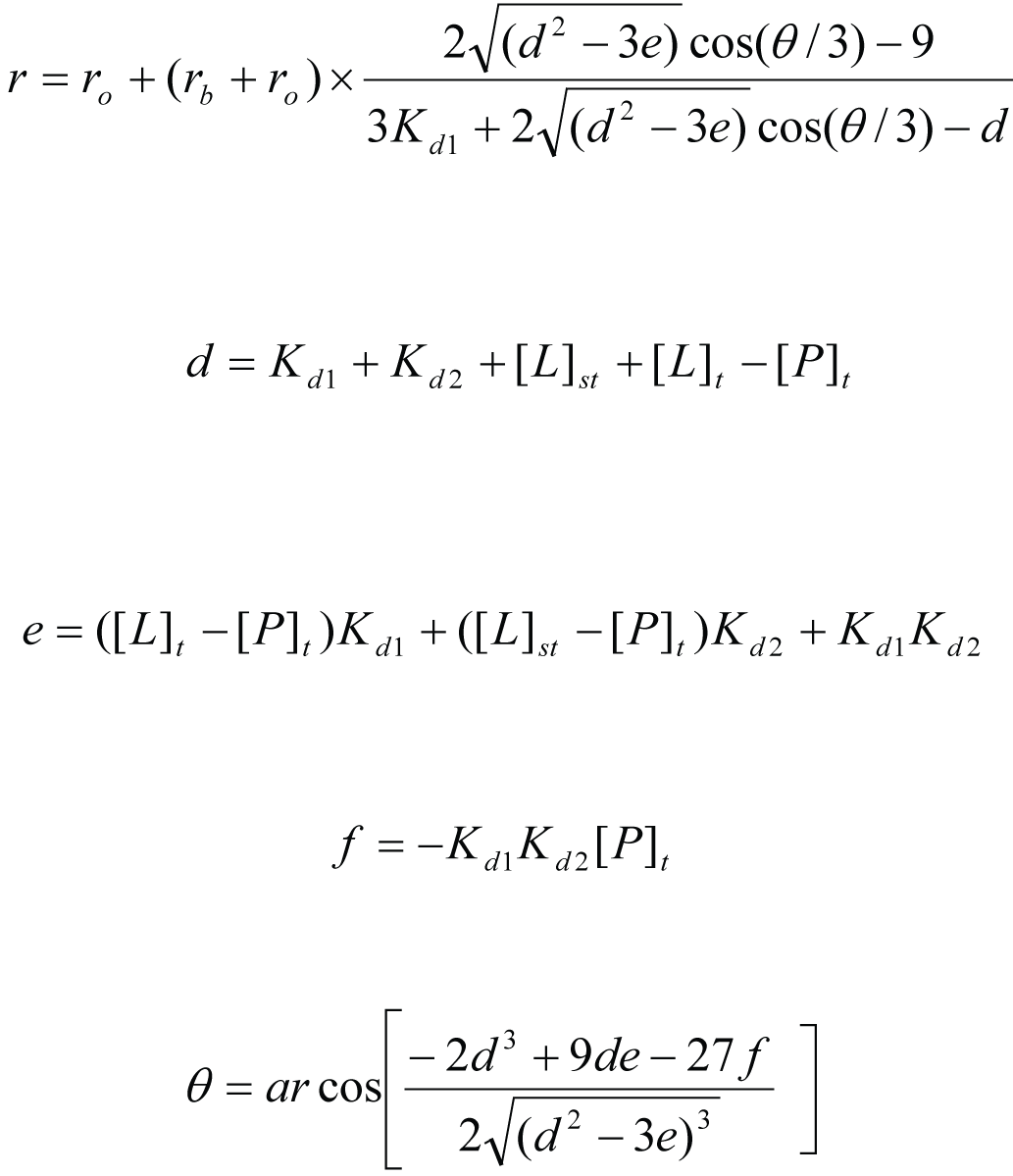


[L]_st_ and [L]_t_ denote labeled ligand and total unlabeled ligand input concentrations, respectively. K_d2_ is the dissociation constant of the interaction between the unlabeled ligand and the protein. In all competition experiments, it is assumed that [P]t > [L]_st_, otherwise considerable amounts of free labeled ligand would always be present and would interfere with measurements. K_d1_ is the apparent K_d_ for the labeled peptide used and has been experimentally determined as described in the previous paragraph. The FAM-labeled peptide was dissolved in dimethyl sulfoxide (DMSO) at 1 mM and diluted into experimental buffer. Readings were carried out with an Envision Multilabel Reader (PerkinElmer). Experiments were carried out in PBS (2.7 mM KCl, 137mM NaCl, 10 mM Na2HPO4 and 2 mM KH2PO4 (pH 7.4)) and 0.01% Tween 20 buffer. All titrations were carried out in triplicate. Curve-fitting was carried out using Prism 4.0 (GraphPad). To validate the fitting of a 1:1 binding model we carefully ensured that the anisotropy value at the beginning of the direct titrations between Mdm2 and the FAM-labeled peptide did not differ significantly from the anisotropy value observed for the free fluorescently labeled peptide. Negative control titrations of the ligands under investigation were also carried out with the fluorescently labeled peptide (in the absence of Mdm2) to ensure no interactions were occurring between the ligands and the FAM-labeled peptide. In addition, we ensured that the final baseline in the competitive titrations did not fall below the anisotropy value for the free FAM-labeled peptide, which would otherwise indicate an unintended interaction between the ligand and the FAM-labeled peptide to be displaced from the Mdm2 binding site.

**Western Blot Analysis**

CHO-K1 cells were seeded the day before of the treatment on a 96 well plate at a concentration of 10000 cells/well in DMEM cell medium 10% FCS (v/v). After an overnight incubation, the cell media in each well was replaced with 90 µl of fresh cell media containing FCS 2% (v/v). 10 µl of compound in 10% DMSO (v/v) in HPLC grade water (reconstituted from DMSO stock solutions) was added for 1 or 24 hrs at the indicated concentrations. The final residual DMSO concentration was 1% (v/v). After the indicated compound incubation times, cells in each well were lysed with 60 µl of lysis buffer (20 mM Hepes pH 7.4, 100 mM NaCl, 5 mM MgCl_2_ , 0.5% NP-40, 1 mM dithiothreitol, protease inhibitor set (cOmplete^™^, Roche) and phosphatase (SigmaAldrich) inhibitor cocktail). Cellular debris was separated by centrifugation, and 30 µl of whole cell extract was resolved on a Biorad polyacrylamide gel (4-20%) according to the manufacturer’s protocol. Western transfer was performed with an Immuno-blot PVDF membrane (Bio-Rad) using a Trans-Blot Turbo system (Bio-Rad).  Antibodies used for western blotting against p53 (1C12) and β-actin were purchased from Cell Sgnalling technology. The 1C12 antibody was used at a 1:2000 dilution. Otherwise western blotting was performed as indicated by manufacturer’s protocol. Western blot images were obtained with an Odyssey Imaging system and were saved as 300 DPI TIFF files. Images were then processed using Adobe Illustrator.

**Peptide Synthesis**

Peptide Synthesis Ramage Chemmatrix resin was obtained from PCAS-Biomatrix (Quebec, Canada). L-amino acids were obtained from Advanced Chemtech (Louisville, KY). Fmoc-threonine, serine, glutamic acid and tyrosine were t-butyl protected and Fmoc-tryptophan was not Boc protected. Unnatural alkenyl amino acids were purchased from OKeanos (Beijing, China). All other solvents and reagents were obtained from Sigma-Aldrich. 1,2-dichloroethane (DCE) was dried overnight over activated molecular sieves and purged with Argon for 30 min prior to use. All other reagents were used as received. The peptides were synthesized by Fmoc chemistry on a Syro II peptide synthesizer (Biotage) at the 0.1 mmol scale using Ramage Chemmatrix resin (0.53 mmol/g). The dry resin was swelled with 1-methyl-2-pyrrolidinone (NMP) before use. The Fmoc protecting group was removed by treatment with 40% piperidine in NMP (3 min) followed by a second treatment with 20% piperidine in NMP (12 min). The Fmoc-protected amino acids (5 equiv.) were coupled using diisopropylcarbodiimide (DIC) as the activating agent (5 equiv.) and 1-aza benzotriazole (HOAt) as the additive in NMP (0.5 M). The coupling time was 90 min for all amino acids except for (S)- N-Fmoc-2-(4'-pentenyl)alanine and (R)-N-Fmoc-2-(7'-octenyl)alanine (S5 and R8 respectively). S5 and R8 (4 equiv.) were manually pre-activated for 7 min and coupled to the peptide resin for two hours. Following deprotection of the final Fmoc group, the peptides were acetylated using a mixture of acetic anhydride/diisopropylethylamine/dimethylformamide (2/2/1) for 60 min. After each coupling, deprotection and acetylation reaction, the resin was thoroughly washed with NMP. Ring-closing metathesis of resin-bound, N-acetylated peptides was performed manually using a 5 mg/mL solution of Grubbs I catalyst (20 mol%) in dry DCE at room temperature under an atmosphere of inert argon (3 x 2 h treatments). After the reaction, the solution was drained, the resin washed with DCE (3 x 1 min), DMSO (1 x 2 h) and methanol (3 x 1 min) then dried in vacuo overnight. Cleavage of the peptide from the resin was achieved using 8 mL of TFA cocktail consisting trifluoroacetic acid/triisopropylsilane/water (95/2.5/2.5) for 2 h followed by filtration and precipitation with diethyl ether. The precipitate was collected by centrifugation, dried and redissolved in a 3:2 mixture of acetonitrile and water. The pure peptides (>90% purity) were obtained by purification using a preparative HPLC system (Agilent) on a Jupiter C12 reversed-phase preparative column (Phenomenex, 4 μm, Proteo 90 Å, 250 x 10 mm). The peptides were characterized by LC-MS. Mass spectra were obtained by electrospray in positive or negative ion mode.

**Supplementary Figures**

**
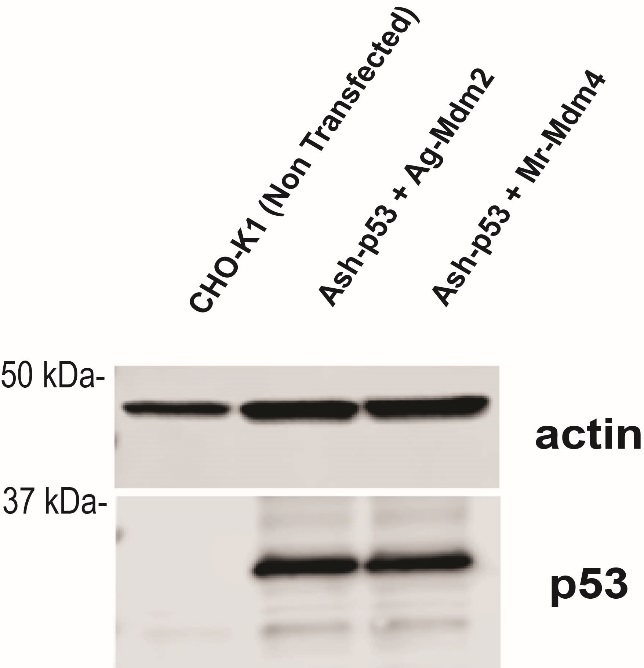
**

**Figure S1:** Western blot analysis of Ash-p53 protein levels in wild-type CHO-K1 cells and CHO-K1 cells stably transfected with the Ash-p53:Ag-Mdm2 and Ash-p53:Mr-Mdm4 FLUOPPI systems. Actin was used as a loading control. The western blot was performed as described in the supplementary methods and materials. The Ag-Mdm2 and Mr-Mdm4 could not be detected due to the lack of suitable specific antibodies. For original unedited western blot images please see **figure S6**.


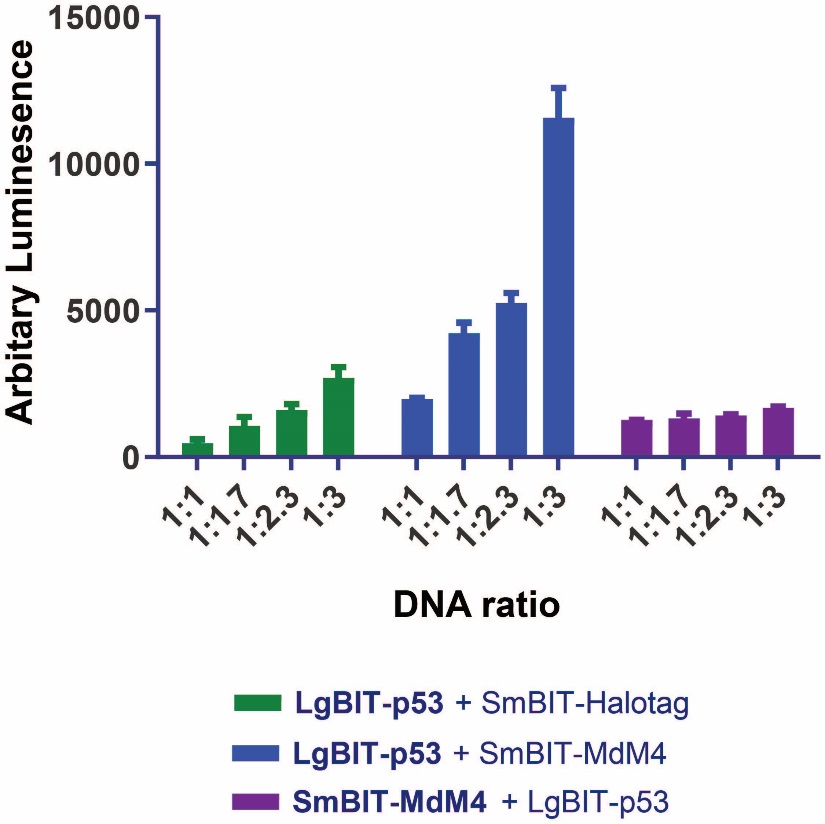


**Figure S2:** The Mdm4 full-length protein was cloned and fused into the smBIT plasmid in the same position and orientation as Mdm2 in the Mdm2:p53 NanoBIT PPI assay system (PROMEGA). The SmBIT-Mdm4 and LgBIT-p53 plasmids were then co-transfected into HEK293 cells at different DNA ratios and assessed for reconstituted luciferase nanoBIT activity. The specificity of the Mdm4:p53 pair was then verified by co-transfecting increasing amounts of the lgBIT-p53 plasmid in relation to the non-binding smBIT-HALO (PROMEGA) construct, a protein that has not been reported to interact with either Mdm2/4. This resulted in negligible increases in luminescence activity compared to the optimal 1:3 co-transfection ratio of LgBIT-p53 to sMBIT-Mdm4.


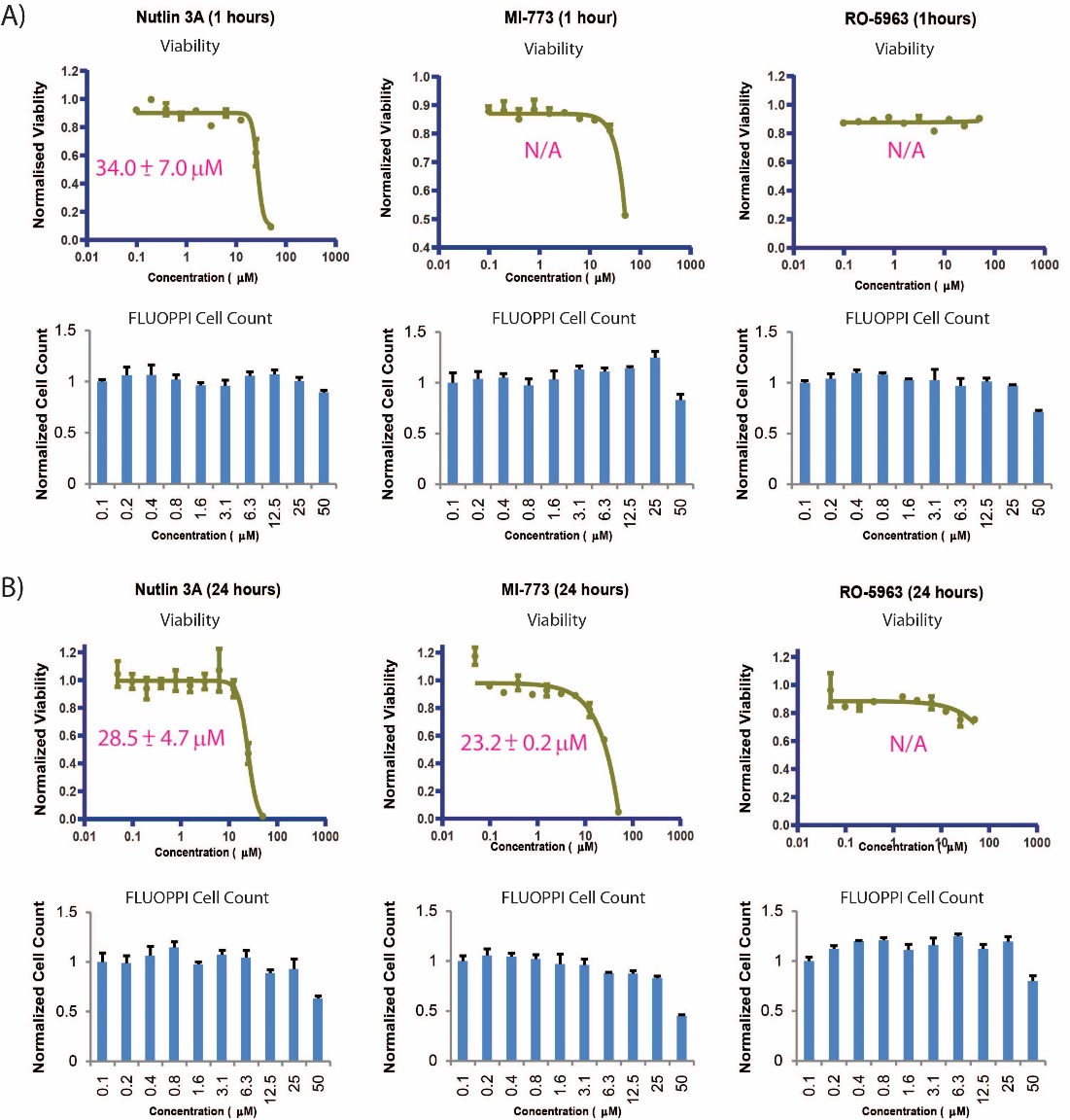


**Figure S3:** CHO-K1 cells were treated with identical titrations of Nutlin 3A, MI-773 and RO-5963 and their effects on viability were assessed after either **A)** 1hours or **B)** 24 hours of treatment. The number of cells used to calculate the FLUOPPI signal (using cells stably transfected with the bimodal p53:Mdm2/4 FLUOPPI system) are shown in bar chart format below the equivalent viability titration. IC_50_ values are indicated next to the relative viability titration. IC_50_ values were derived from the individual titration using a 4-parameter curve fit. Non-linear regression analysis to fit the curve was performed in Prism (Graphpad). Experiments were performed in DMEM cell media containing 10% (v/v) FCS

­­­
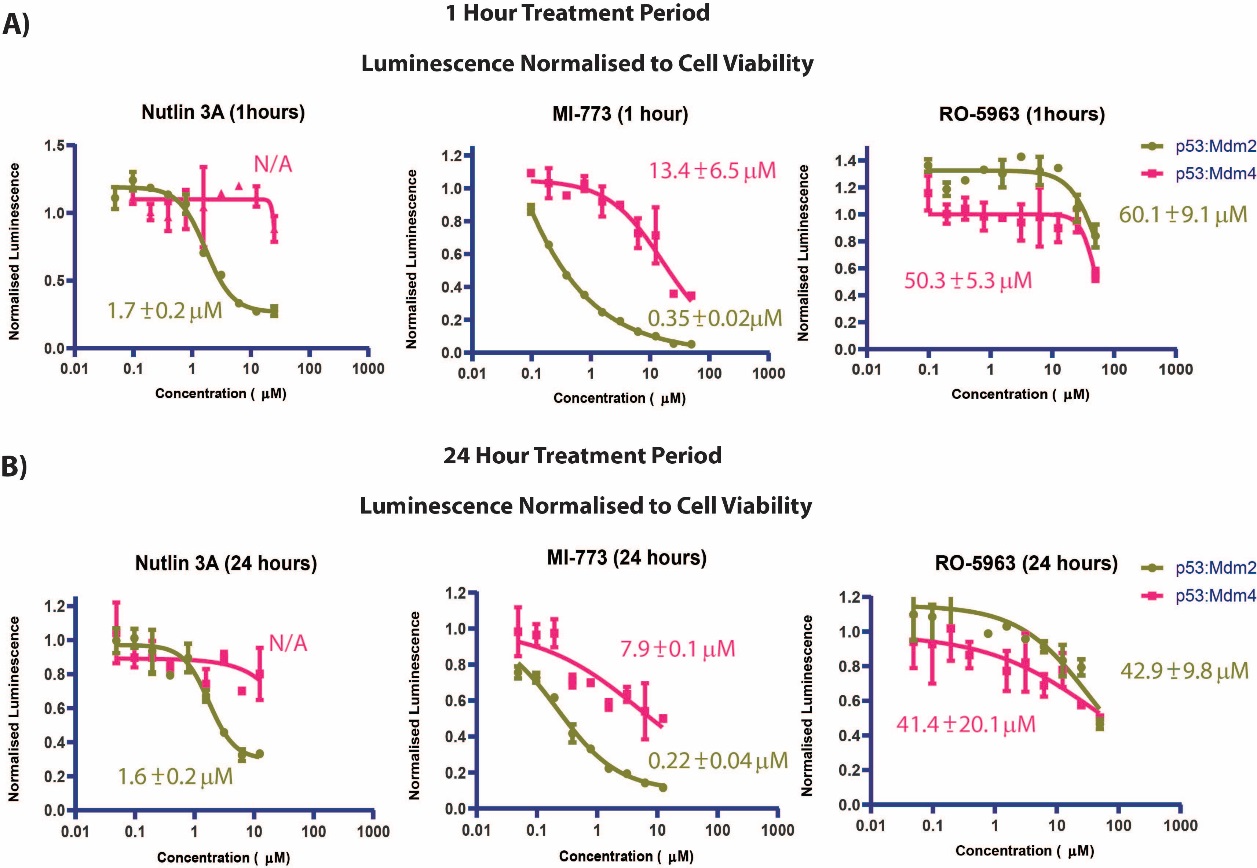


**Figure S4:** Titrations of the Mdm2 specific small molecule inhibitors Nutlin 3A and MI-773, and the small molecule Mdm2/Mdm4 dual inhibitor RO-5963, against CHO-K1 cells transiently transfected with either the Mdm2:p53 or Mdm4:p53 NanoBIT system, which have been normalised to cellular viability (**figure S2**). Additionally data points that decrease in in cellular viability greater than 25% were excluded. Titrations were performed for either **A)** 1hours or **B)** 24 hours. Experiments were performed in DMEM cell media containing 10% (v/v) FCS


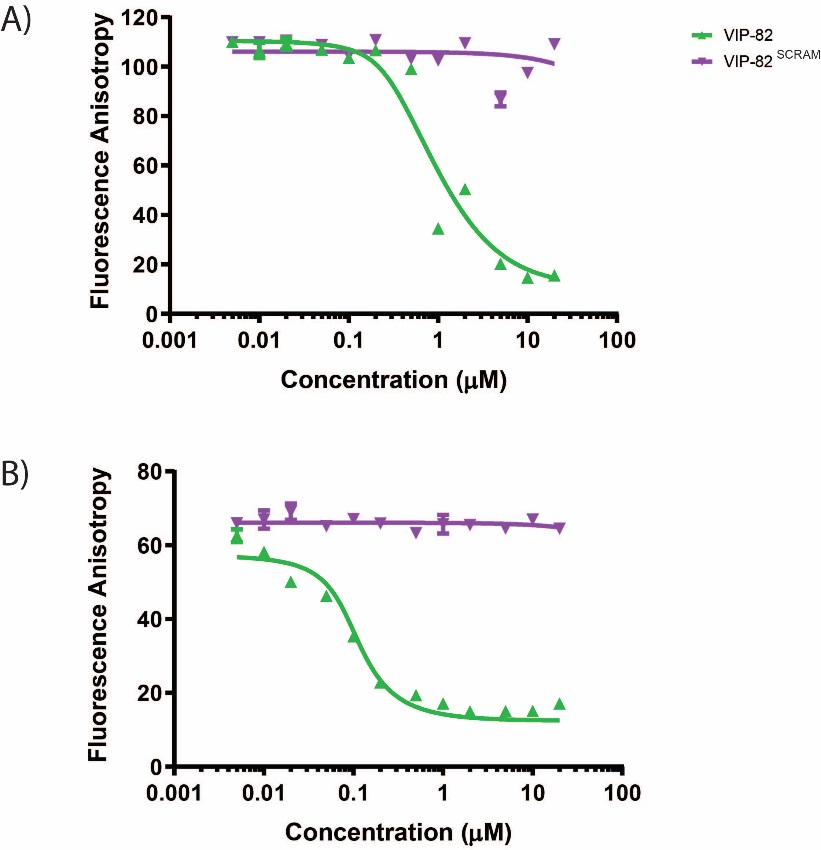


**Figure S5:** VIP-82 and VIP-82^SCRAM^ were both assessed for their ability to interact with the n-terminal domains of **A)** Mdm2 and **B)** Mdm4 using two independent fluorescence anisotropy based competition assays (see methods and material). VIP-82 bound Mdm2 and Mdm4 with K_d_s of 37.3 ± 6.2 and 7.3 ± 1.0, respectively. In contrast VIP-82 was unable to interact specifically with either Mdm2 or Mdm4.

**
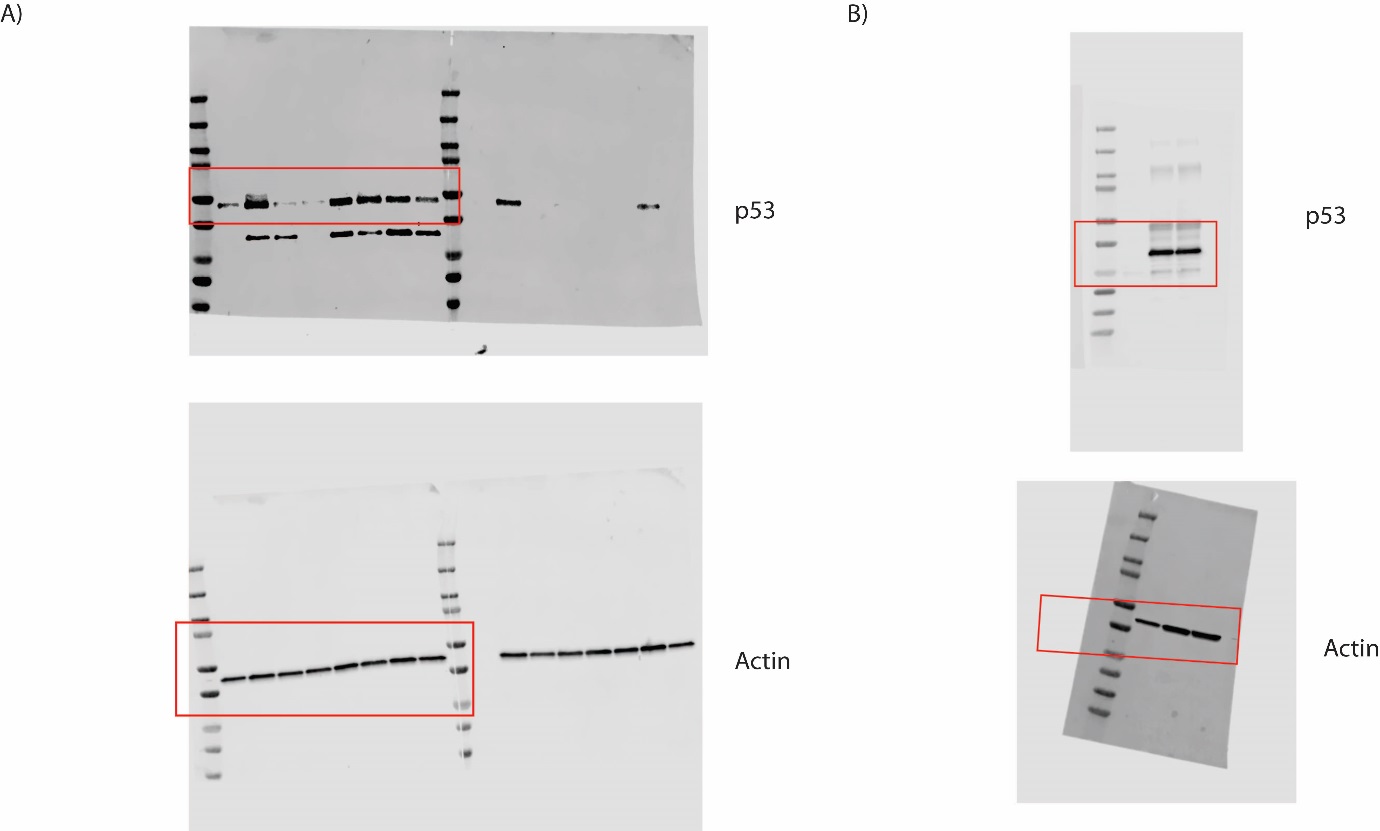
**

**Figure S6:** Original unedited western blots. Images were acquired using an Odyssey Imaging system and were saved as 300 DPI TIFF files. Images were then processed using Adobe Illustrator. **A)** Western blots that were used to construct figure 3 in main manuscript. **B)** Western blots that were used to construct figure S1. Red boxes delineate section of western blot that was used for figure construction.
